# Gold Nanostars as Multifunctional Platforms in Cancer Imaging and Therapy: Toward Radiotheranostic Applications

**DOI:** 10.3390/ph19091476

**Published:** 2026-09-17

**Authors:** Ghazal Basirinia, Muhammad Ali, Albert Comelli, Ayesha Arif, Pierpaolo Alongi, Domenico Di Raimondo, Antonino Tuttolomondo, Viviana Benfante

**Affiliations:** 1 Ri.MED Foundation, Via Bandiera 11, 90133 Palermo, Italy; gbasirinia@fondazionerimed.com (G.B.); amuhammad@fondazionerimed.com (M.A.); aarif@fondazionerimed.com (A.A.); 2 Department of Health Promotion, Mother and Child Care, Internal Medicine and Medical Specialties, Molecular and Clinical Medicine, University of Palermo, 90127 Palermo, Italy; domenico.diraimondo@unipa.it (D.D.R.); bruno.tuttolomondo@unipa.it (A.T.); 3 Department of Biomedicine, Neuroscience and Advanced Diagnostics (BiND), University of Palermo, 90127 Palermo, Italy; pierpaolo.alongi@unipa.it; 4 Advanced Diagnostic Imaging-INNOVA Project, Department of Radiological Sciences, A.R.N.A.S. Civico Di Cristina e Benfratelli Hospitals, P.zza N. Leotta 4, 90127 Palermo, Italy; viviana.benfante@arnascivico.it

**Keywords:** gold nanostar, cancer therapy, radionuclide therapy, theranostic, nanomedicine, nanotechnology

## Abstract

Gold nanostars (AuNSs) have emerged as promising platforms for cancer theranostics due to their distinctive optical and physicochemical properties, including strong plasmon resonance, tunable structure, and high photothermal conversion efficiency. These features support their use in various imaging modalities such as surface-enhanced Raman scattering (SERS), fluorescence, computed tomography (CT), magnetic resonance imaging (MRI), and photoacoustic imaging as well as in therapies like photothermal, photodynamic, and radiotheranostic treatments. The ability of GNS-based systems to integrate diagnostic and therapeutic functions in a single way facilitate real-time treatment monitoring and improves therapeutic accuracy. Furthermore, surface functionalization and the development of hybrid nanostructures can enhance tumor-targeting capability, biocompatibility, and performance in biological environments. However, several challenges such as limited tumor specificity, potential toxicity, complex interactions in biological systems, and difficulties in large-scale synthesis remain and limit their preclinical translation. Therefore, future efforts on optimizing nanoparticle stability, biodistribution, and safety while incorporating advanced strategies would support their preclinical and anticipated clinical application. In this review, we focus on the design, functionalization, and biomedical applications of gold nanostars in cancer imaging and therapy with future perspective in radiotheranostic strategies.

## 1. Introduction

Despite advances in treatment for complex and multifactorial disease like cancer [1], the accurate evaluation of therapeutic response remains a significant challenge. Conventional assessment methods, such as the Response Evaluation Criteria in Solid Tumors (RECIST), rely primarily on changes in tumor size measured through anatomical imaging modalities, including computed tomography (CT) and magnetic resonance imaging (MRI). However, these approaches often fail to capture early or subtle biological responses, particularly in the case of targeted therapies, where tumor shrinkage may not directly correlate with therapeutic efficacy. So, there is an increasing demand for advanced imaging strategies capable of providing functional and molecular level insights. Molecular imaging techniques have therefore gained prominence as they enable the visualization, characterization, and quantification of biological processes at cellular and subcellular levels, offering a more accurate and timely evaluation of treatment outcomes [2].

Theranostics integrating diagnostic and therapeutic functionalities within a single platform has gained substantial attention in modern medicine. By integrating molecular imaging with targeted treatment, theranostic approaches allow for patient-specific intervention, real-time monitoring, and improved precision in disease management. This approach has been further strengthened by advances in nanotechnology and molecular biology, which have facilitated the design of multifunctional systems capable of both detecting and treating disease at the molecular scale. A significant extension of this concept is radiotheranostics, a rapidly evolving field that employs radiolabeled agents for both diagnostic imaging and targeted radionuclide therapy. Examples of clinically approved radiotheranostic therapies, such as iodine-131 therapy for thyroid cancer, radium-223 therapy for bone metastases, lutetium-177 DOTATATE, and lutetium-177 PSMA for prostate cancer therapy, demonstrate the transformative clinical potential of this strategy. Despite these advances, challenges related to dosimetry optimization, appropriate patient selection, and logistical implementation remain to be addressed [3,4].

Within this rapidly advancing landscape, nanomaterials, particularly gold-based nanostructures, are considered as highly versatile platforms for cancer theranostics. Gold nanoparticles (AuNPs) exhibit unique physicochemical properties, such as tunable optical behavior, high biocompatibility, and ease of surface functionalization, making them ideal candidates for both imaging, drug delivery and combined therapeutic applications. In Comparison with conventional therapeutic approaches such as surgery and chemotherapy, appropriately engineered AuNPs may provide extended circulation times, enhanced tumor targeting through receptor-mediated endocytosis, and improved performance as an imaging contrast agent, particularly in techniques such as CT. In addition, environmentally friendly “green” synthesis methods, which use biological precursors, have enabled scalable and sustainable production of AuNPs. Nevertheless their biological effects remain dependent on parameters such as particle size, morphology, surface chemistry, and reactivity [5].

**Figure 1:**
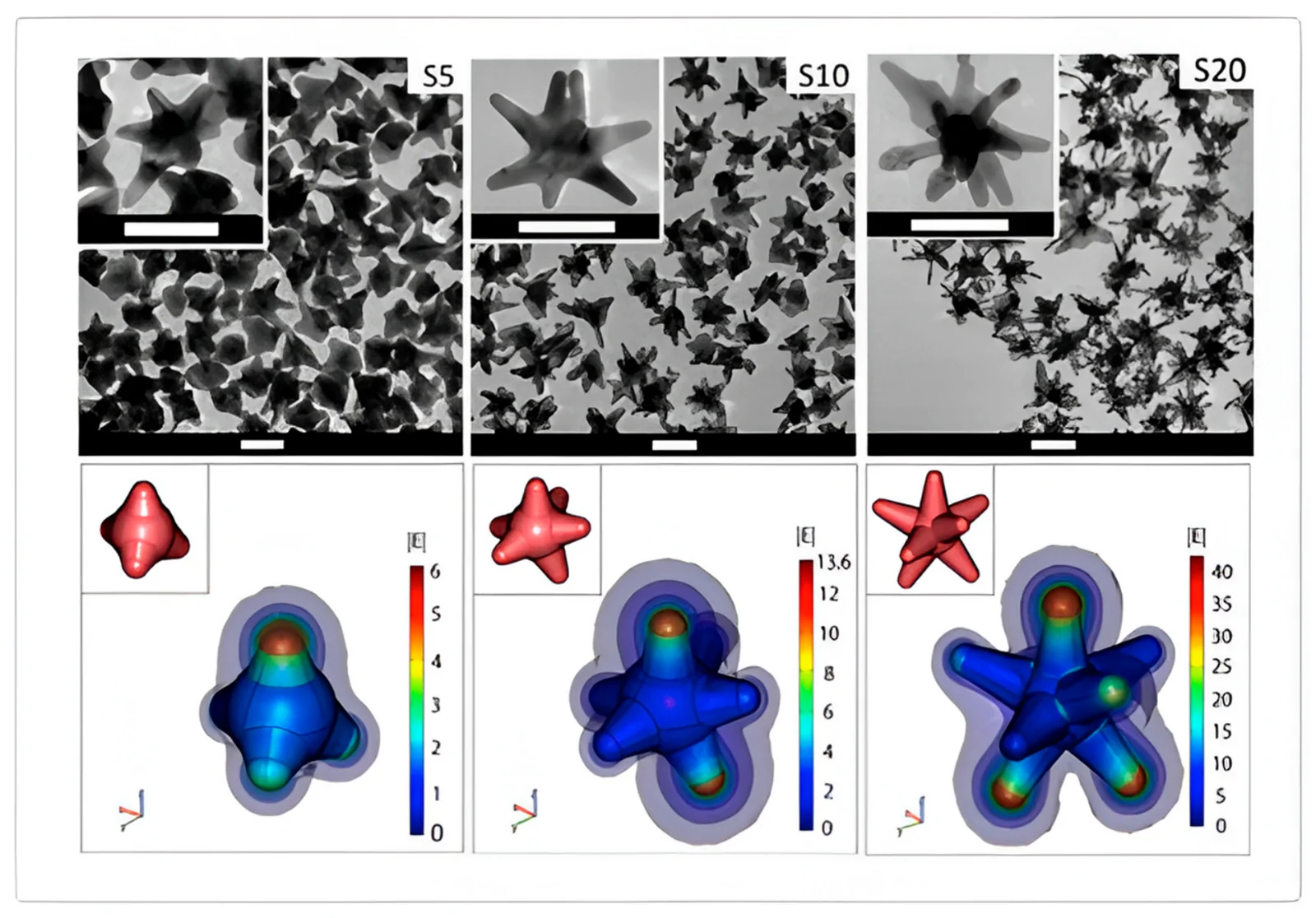
Gold nanostars illustrate unique optical tunability, as their sharp multi-branched geometry produces a “lightning rod” effect, significantly boosting local electromagnetic fields and providing highly sensitive SERS-based detection and bioimaging which exhibit (**Top**) transmission electron microscope (TEM) images of gold nanostars (AuNSs) with varying branch numbers (scale bar: 50 nm). (**Bottom**) Simulated electromagnetic field distribution (|E|) around AuNSs under illumination (λ = 800 nm), showing strong field enhancement at the sharp tips due to the “lightning rod” effect. Insets present the corresponding 3D structures (not to scale). Reprinted from ref. [6] and adapted from ref. [7] under CC BY license.

Among the different range of plasmonic nanostructures, including gold nanospheres, nanoshells, nanorods, and nanocages, gold nanostars (AuNSs) have respected particular attention due to their characteristic multi-branched morphology. Different from conventional spherical nanoparticles, AuNSs show remarkable optical tunability that can be precisely controlled through subtle variations in their geometric features. Especially, the presence of multiple sharp branches generates a pronounced “lightning rod” effect, significantly amplifying the local electromagnetic (EM) field at the nanoparticle tips (Figure 1). This enhancement leads to strong localized surface plasmon resonance (LSPR) and contributes highly intensified signals in techniques such as surface-enhanced Raman scattering (SERS), consequently enabling ultrasensitive detection and high-resolution bioimaging [6].

This unique geometry generates intense LSPR at the tips, leading to exceptional light absorption and highly efficient photothermal conversion. These properties make AuNSs especially suitable for photothermal therapy (PTT), where localized heat generation can selectively ablate tumor tissues. Importantly, GNS-mediated PTT has also been shown to induce immunogenic cell death, thereby enhancing antitumor immune responses. When combined with immune checkpoint inhibitors, this approach can overcome tumor-induced immunosuppression, suppress metastasis, and even induce long-term systemic immunity, resembling a therapeutic “cancer vaccine” effect [8].

Beyond photothermal applications, gold nanostars demonstrate remarkable versatility across multiple biomedical domains, including biosensing, targeted drug delivery, and advanced imaging techniques such as SERS, two-photon photoluminescence, CT, and MRI. Their tip-enhanced electromagnetic fields enable ultrasensitive detection and imaging, while their surface can be readily functionalized with targeting ligands, drugs, or radionuclides. These multifunctional capabilities position AuNSs as a powerful platform for next-generation theranostic systems [9,10].

Whereas many gold nanostar studies mainly focus on photothermal therapy, optical imaging, or drug delivery, the perspective of this work is to develop gold nanostars as a radiotheranostic nanoplatform. Gold nanostars are not only plasmonic photothermal agents, but also high-Z nanostructures with potential to enhance radiation response and to serve as carriers for diagnostic or therapeutic radionuclides [11]. Radiolabeling approaches have advanced from conventional chelator-based methods to chelator-free, doping, surface adsorption, and encapsulation strategies, which improve radiochemical efficiency, pharmacokinetic behavior, and targeted delivery, while enabling the use of both conventional and emerging radionuclides, including alpha emitters. Combining radionuclides with chemotherapy may also enhance treatment through synergistic effects but most evidence remains in preclinical study derived from animal studies with limited clinical trials. Major challenges in standardized production, regulation, long-term safety, and clinical outcome remain, so radiolabeled nanomaterials are promising, but their near-term clinical role in radiopharmacy remains uncertain [12]. Recent advances in radioligand design include a transition from agonist-based compounds toward antagonists, improved peptide structures and the use of new therapeutic radionuclides such as ^161^Tb, ^225^Ac, and ^212^Pb to potentially enhance efficacy and reduce toxicity compared with ^177^Lu. Also, paired isotopes are increasingly used because they provide similar targeting and biodistribution for both imaging and therapy. In addition, radiohybrid systems offer different radionuclides to be incorporated within the same molecular platform, increasing flexibility in radiotheranostic design [13]. Thus, our novelty lies in positioning optimized and stabilized gold nanostars as a radiotheranostic platform, where their plasmonic star-shaped architecture can support photothermal therapy and imaging, while their gold-based high-Z composition and potential radiolabeling capacity may enable radiation enhancement, SPECT/PET-guided biodistribution analysis, dosimetry, and radionuclide-based therapeutic strategies. In this review, we focus on the emerging applications of gold nanostars in cancer imaging and therapy, with particular emphasis on their role in advanced theranostic strategies and exploring their integration with radiotheranostic approaches, highlighting their potential as platforms for radionuclide delivery in future.

## 2. Methods

**Figure 2:**
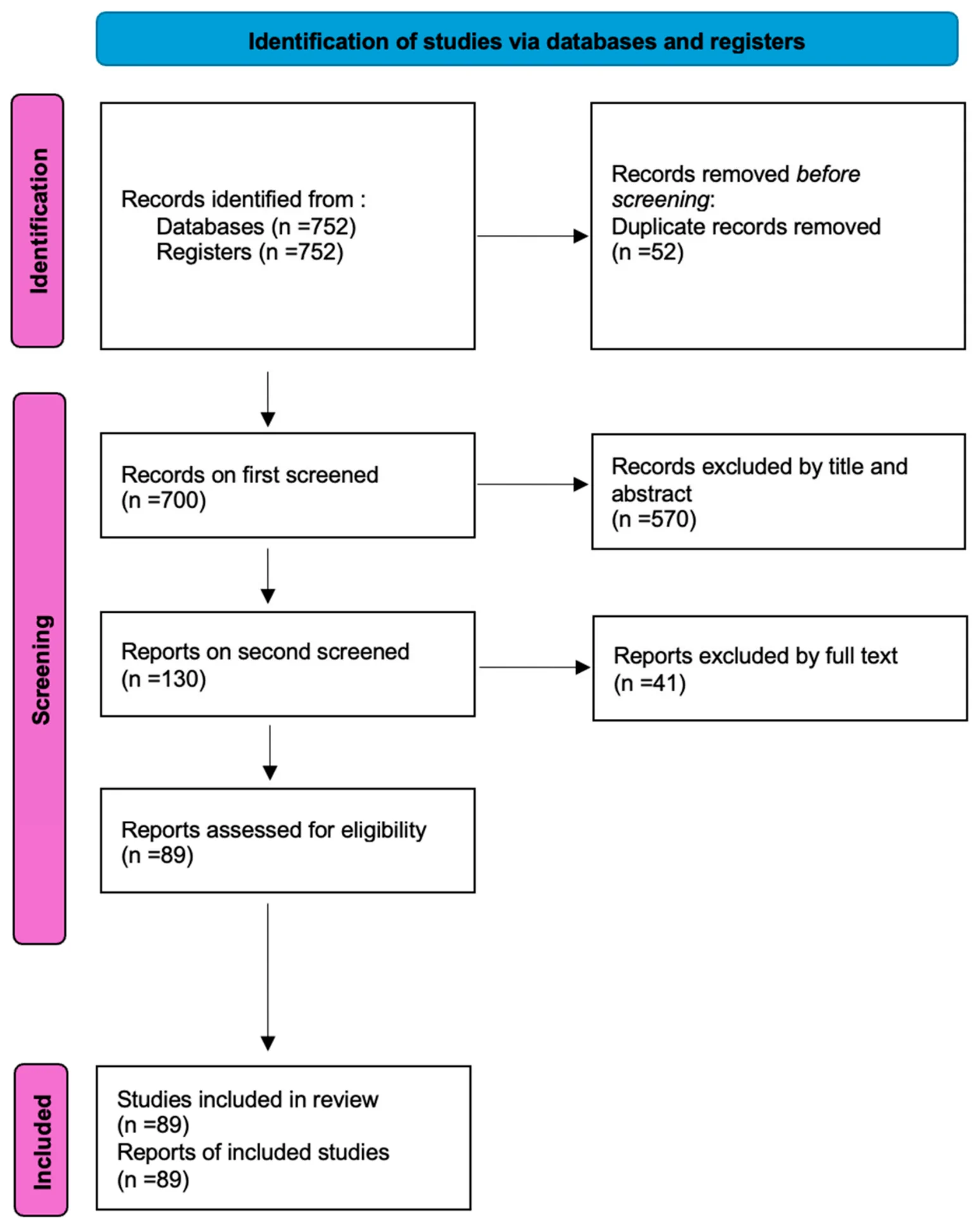
Flowchart of the methodology.

This review utilized scientific databases, including PubChem, PubMed, Scopus, and Google Scholar. The search included combinations of keywords such as (Gold nanostar) AND (IR OR ionizing radiation), (Gold nanostar AND Cancer therapy), (Nanostar AND Theranostics), Nanomedicine, Nanotechnology, Radionuclide Therapy, Radiotherapy and Combined Therapy. The review included studies published between 2012 and 2026, focused on characterization and functionalization of gold nanostars and their preclinical applications for cancer detection, diagnosis, and therapy, and their theranostic use. Therapeutic approaches are including photothermal therapy, chemotherapy, radionuclide therapy, and combination therapy in various cancers. These studies were selected based on their experimental design and reported outcomes related to the use of gold nanostars. The screening process was performed by the authors on title and abstracts to assess their relevance to the topic, and studies that did not focus on gold nanostars characterized or functionalized for various cancers, diagnosis and combined therapy or theranostic approaches, preclinical assays, in vitro, and in vivo were excluded. Original research and review articles in the English language were included, while articles written in non-English languages were excluded. Initially, 752 articles were identified via all databases and after eliminating duplicate articles, 700 unique articles remained. This filtering process resulted in 570 excluded articles. Following this, a total of 89 studies remained, which were evaluated based on our critical topics, including gold nanostar synthesis and characterization, functionalization, cancer imaging and diagnosis, photothermal therapy, chemotherapy, radiotherapy, radionuclide therapy, and combined theranostic strategies. This is summarized in Figure 2.

## 3. Nanostars Used for Imaging

A summary of the gold nanostar-based imaging studies discussed in the following subsections is provided in Table 1.

### 3.1. General Imaging Probe

Bibikova et al. reported a controlled synthesis strategy of plasmon-resonant gold nanostars (AuNSs) with tunable optical properties that were optimized for different biphotonic imaging modalities. Through systematic variation of the size and concentration of spherical seeds, they tailored nanostar dimensions precisely, along with plasmon-resonant peak positions and scattering-to-absorption ratios. The produced nanostars that showed size-dependent performance as contrast agents for optical coherence tomography, Doppler optical coherence tomography (OCT), and confocal laser scanning microscopy. Imaging experiments in living cells exhibited effective visualization of nanostars with strong signal intensity, which was correlated to particle size and the generating wavelength of the imaging system. These results indicate the importance of matching the optical properties of nanostars to specific imaging modalities, and support their use as versatile contrast agents for visible and near-infrared biphotonic applications [14].

Another study by Zhang et al. described a simple surfactant-free method for highly branched gold nanostar synthesis and morphology controlled for biomedical applications. The one-pot seedless synthesis method that they employed used gallic acid as a natural reducing and stabilizing agent to systematically change nanostar size and shape by varying the gallic acid concentration. Under optimal conditions gold nanostars with an average size of approximately 100 nm exhibited strong near-infrared absorption, high photothermal conversion efficiency, and favorable biocompatibility. Moreover, experiments in living cancer cells confirmed successful photoacoustic imaging and effective photothermal therapy. These results indicate that facile, green-synthesized gold nanostars represent a promising platform for future integrated diagnosis and photothermal treatment of cancer [15].

Yang Liu et al. investigated a multifunctional gold nanostar-based theranostic platform designed for combined cancer diagnosis and therapy. The developed gold nanostar probes supported multimodal imaging, including SERS detection, CT imaging, and two-photon luminescence, alongside efficient photothermal therapy. Comprehensive biodistribution and imaging analyses revealed size-dependent tumor uptake and penetration, with smaller nanostars achieving higher intratumoral distribution. Also, the probes demonstrated strong photothermal conversion efficiency and efficient tumor ablation under clinically relevant laser conditions. The findings show the potential of gold nanostars as versatile nanoprobes for in vivo biosensing, image-guided surgery, and photothermal cancer therapy [16].

Liang et al. improved a model of CD44v6 monoclonal antibody-conjugated gold nanostars for use in targeted photoacoustic imaging and plasmonic therapy. They reported the development of PEGylated gold nanostar-based nanoprobes functionalized with CD44v6 monoclonal antibodies for both imaging and photothermal therapy of gastric cancer stem cells. The nanoprobes showed high specificity in vitro on CD44v6-expressing spheroid colonies and achieved efficient photothermal ablation at low laser power. In orthotopic and subcutaneous gastric cancer models, the nanoprobes accumulate actively in tumor-associated vasculature, boosting photoacoustic imaging-guided therapy. After near-infrared laser irradiation, they observed significant tumor growth suppression and extended survival. These findings indicate the potential of CD44v6-targeted gold nanostar nanoprobes as a great strategy for selective gastric cancer stem cell imaging and photothermal treatment [17].

**Figure 3:**
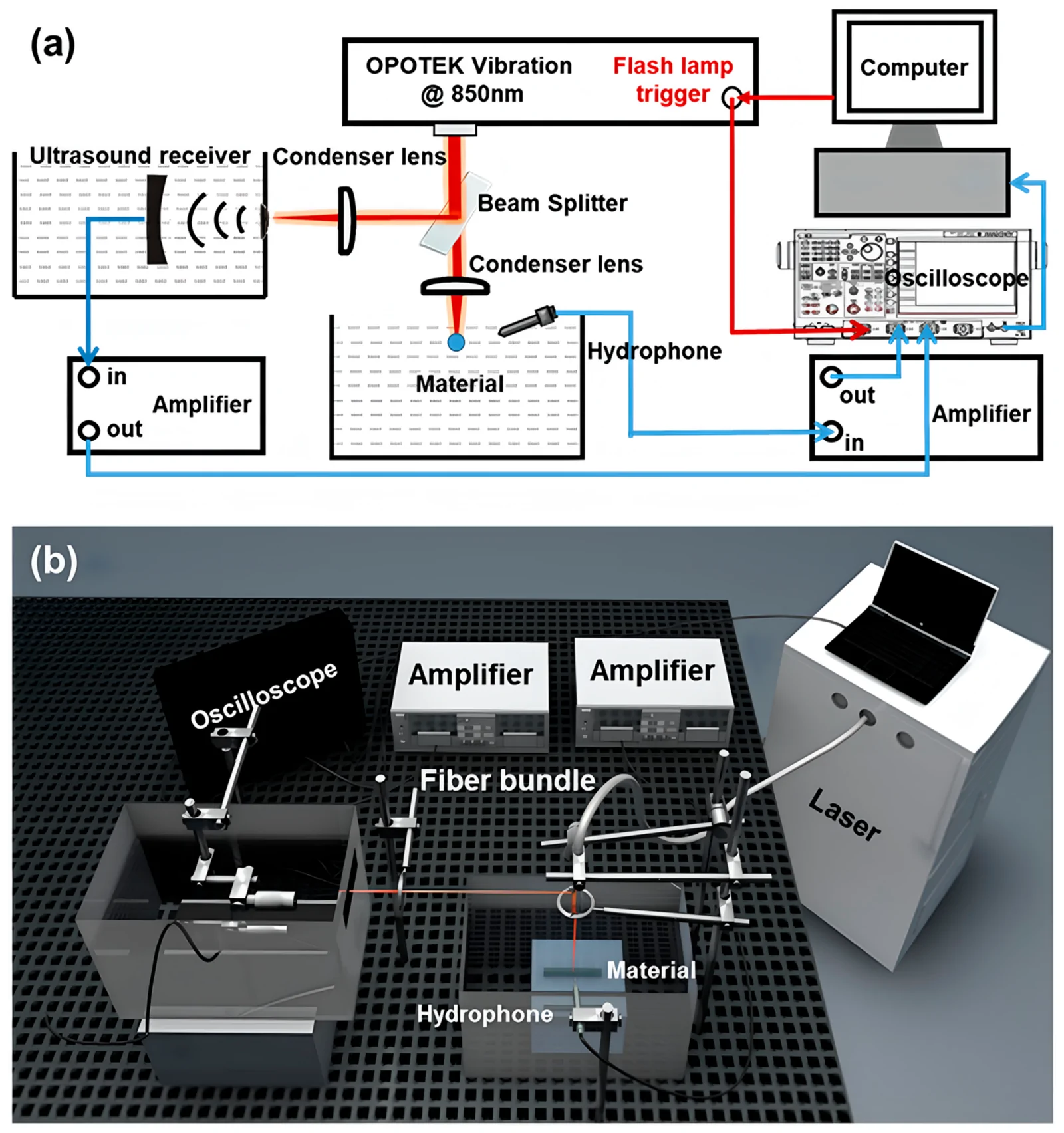
In vitro experimental configuration. (**a**) In vitro photoacoustic experiment’s schematic diagram. (**b**) Schematic representation of 3D in vitro photoacoustic experiment. The laser system was synchronized with the oscilloscope. Reprinted from ref. [19]. Copyright 2021, Photoacoustics. This article is an open-access article distributed under the terms and conditions of the Creative Commons Attribution (CC BY-NC-ND 4.0) license.

Vijay Raghavan et al. developed a dual plasmonic gold nanostar platform engineered as a single component theranostic agent for combined photoacoustic imaging and photothermal therapy. The synthesis process involved a simple chemical reduction method, and the nanostars showed strong optical absorption in the near-infrared region, supporting both efficient photoacoustic signal generation and heat conversion under 1064 nm laser irradiation. Longer wavelength use facilitated deeper tissue penetration for both imaging and therapeutic applications, while silica coating further enhanced imaging performance. In vivo experiments in tumor-bearing mice demonstrated substantial tumor ablation following photothermal treatment. All these findings highlight dual plasmonic gold nanostars as promising multimodal nanoconstructs for integrated diagnosis and treatment of cancer [18]. Furthermore, Chen et al. showed photostability improvement of silica-coated gold nanostars for photoacoustic imaging (PAI) and guided photothermal therapy (Figure 3). They synthesized gold nanostars using a surfactant-free strategy and encapsulated them within a silica shell to prevent laser-induced morphological reshaping. The gold nanostars’ performance was evaluated in vitro and in vivo, using mesenchymal stromal cells (MSCs) and tumor-bearing nude mice. Silica-coated nanostars, compared with uncoated AuNSs, exhibited almost threefold greater photoacoustic signal stability, illustrating enhanced resistance to structural shifting under laser irradiation. By preserving plasmonic features and maintaining consistent photoacoustic contrast, silica encapsulation significantly improved GNS-based PAI reliability and safety, supporting their application as durable contrast agents for imaging and photoacoustic-guided theranostics in cancer [19].

Wang et al. reported a highly efficient photothermal theranostic platform based on a gold nanostar–polyaniline core–shell nanocomposite for photoacoustic imaging guided photothermal therapy. By synergistically integrating polyaniline with gold nanostars, the nanocomposite achieves an exceptionally high photothermal conversion efficiency, enabling effective tumor ablation at low material dosage. Surface modification with hyaluronic acid provides active tumor targeting while preserving photothermal performance. The nanoprobes exhibit good biocompatibility and strong therapeutic efficacy against triple-negative breast cancer in both in vitro and in vivo models. Notably, significant tumor suppression is achieved with a single low-dose injection, highlighting the potential of this nanocomposite for possible safe and efficient photothermal cancer therapy and future translational applications [20].

Ping Hu et al. worked on folic acid-conjugated gold nanostars for use in computed tomography imaging and combined photothermal–radiation therapy. Folic acid-functionalized PEGylated gold nanostars (GNS-FA) as multifunctional theranostic agents were engineered for both tumor-targeted CT imaging and synergistic treatment by photothermal–radiotherapy. The nanostars showed good biocompatibility and served as effective CT contrast agents. Also, the properties of gold, such as unique optical and X-ray attenuation, enhanced photothermal transducers and radiosensitizers. In vitro tests on HeLa cells demonstrated that the combined PTT/RT treatment produced higher anticancer efficacy compared to photothermal therapy or radiotherapy alone. These results highlight the potential of GNS-FA as a valuable nanoplatform for CT imaging-guided combined cancer therapy [21].

Wang et al. investigated a strategy of using gold nanostars as multifunctional theranostic agents for cancer imaging and treatment. In vitro experiments on breast cancer cells (MCF-7) demonstrated efficient localization of GNSTs within both cytoplasm and nuclei in cancer cells. When near-infrared (NIR) irradiation happened, the nanostars induced combined photothermal and photodynamic effects through generating heat and reactive oxygen species (ROS). The results showed effective DNA damage, G0/G1 cell-cycle arrest, glutathione depletion, and apoptosis increasing. In vivo tests with intratumoral administration of GNSTs were performed and followed by NIR irradiation that significantly suppressed tumor growth. Furthermore, the nanostars exhibited strong X-ray attenuation properties, enabling their use as X-ray imaging contrast agents. All findings indicate the potential of gold nanostars as multifunctional platforms integrating diagnostic imaging, photothermal therapy, and photodynamic therapy for cancer theranostics [22]. Also, Espinosa et al. showed the impacts of gold nanostar internalization in cancer cells and tumoral environments based on plasmonic thermal fingerprints in photothermal evaluation in vitro and in vivo. They conducted in vitro and in vivo experiments on a prostate cancer cell line (PC3) and PC3 tumors in mice with different sizes of gold nanostars upon various wavelengths. The results showed these parameters have a significant impact on heating efficiency. Thus, the plasmonic heating response acts as a signature for gold nanoparticles intracellular tracking, which illustrates improvements in the role of nanoparticle-mediated photothermal therapies [23].

### 3.2. Raman-SERS Imaging—Fluorescence and Confocal Microscopy

Luca et al. and collogues with SERS-active gold nanostars offered a nanoplatform for combined bioimaging and drug delivery. In this study, star-shaped gold nanoparticles were functionalized with doxorubicin and coated with immunomimetic thiolated PEG; the results showed good stability and low-cytotoxic conjugates with strong Raman enhancement. The close adsorption of doxorubicin enables in vivo SERS-based intracellular tracking, while prolonged laser irradiation triggers thermally driven drug release. Use of both SERS with confocal microscopy allowed real-time monitoring of nanoparticle localization and drug delivery in living cells [24].

Juang Wang et al. presented a multifunctional gold nanostar-based theranostic nanocomposite designed for tumor-targeted photothermal therapy with self-monitoring capability. By integrating gold nanostars and a fluorescent dye through a caspase-3-responsive peptide, the system enables fluorescence quenching under normal conditions and signal recovery upon apoptosis induction. Folic acid functionalization enhances selective uptake by cancer cells, while photothermal irradiation triggers effective tumor cell killing via apoptosis. In addition, real-time feedback on therapeutic efficacy provided by subsequent activation of caspase-3 restores fluorescence. This method offers a good platform for precise photothermal cancer therapy combined with simultaneous apoptosis imaging and treatment monitoring [25].

Nerigiz et al. aimed to develop a possible safe, multifunctional nanoplatform for image-guided and externally triggered cancer therapy. They engineered hybrid nanopatches composed of graphene oxide and gold nanostars with tunable near-infrared plasmonic resonance using a biocompatible synthesis method. Cellular uptake in breast cancer epithelial cells was investigated using Raman imaging, transmission electron microscopy, and elemental analysis. The hybrid nanopatches exhibited excellent biocompatibility, minimal cytotoxicity, and significantly enhanced photothermal efficiency at low dosages and laser power compared with individual components. Additionally, strong Raman scattering enabled effective imaging capability. These results highlight the potential of graphene oxide–gold nanostar hybrids for future applications in bioimaging, diagnostics, and image-guided photothermal cancer therapy [26].

Leyong Zeng et al. introduced a multifunctional Ag core–Au shell gold nanostar coupled with a Raman reporter to enhance SERS imaging and near-infrared-triggered photothermal therapy for breast cancer. The Au shell coating created effective biocompatibility while preserving strong plasmonic and photothermal features. The two-step DTTC coupling strategy significantly enhanced Raman signal intensity and stability, enabling sensitive SERS imaging both in vitro and in vivo. The nanostars demonstrated excellent photothermal performance, leading to superior tumor eradication under NIR laser irradiation with minimal toxicity. These Ag@Au-DTTC nanostars designed a great low-risk theranostic agents for combined imaging and photothermal therapy in cancer [27].

Antoine D’Hollander et al. addressed a critical limitation by mitigating protein corona formation in nanoparticle-based cancer theranostics, which often compromises active targeting in biological environments. Through selective chemical blocking of gold nanostar surface ligands using 2-mercaptoethanol, they reduced corona thickness successfully while preserving target-site specificity. Conjugation of a HER2-specific nanobody able to selectively recognize HER2-positive tumors was illustrated both in vitro and in vivo using darkfield microscopy and photoacoustic imaging. The results highlight gold nanostars as perfect theranostic agents with preserved targeting specificity in complex biological systems, and suggest an effective approach for enhancing translational potential of targeted nanomedicine [28].

Antoine D’Hollander et al. also conducted research on biocompatible gold nanostars that have been explored as highly sensitive SERS contrast agents for cancer detection. Their efficient uptake by ovarian cancer cells (SKOV3) enabled intracellular localization and imaging through SERS, while systemic administration in xenograft-bearing mice resulted in detectable tumor-associated signals in vivo. The accumulation of nanostars in tumors was confirmed by ex vivo SERS measurements and dark-field microscopy, demonstrating their promise as SERS-based imaging probes and potential theranostic agents for cancer management [29].

Depciuch et al. developed and evaluated gold nanostars with tunable branch lengths as photothermal agents for cancer therapy. TEM, selected area diffraction (SEAD) and X-ray diffraction (X-Ray) analyses confirmed the presence of highly crystalline nanostars with similar core sizes (140–146 nm), while branch lengths increased from 193 nm (AuS1) to 356 nm (AuS4) depending on reducer concentration. Increasing arm length produced a red shift in the plasmon resonance peak from 640 to 770 nm and significantly enhanced photothermal conversion efficiency and heat generation under laser irradiation. The photothermal performance was assessed in SW480 and SW620 colon cancer cell lines, where nanostars with the longest branches (AuS4) exhibited the greatest therapeutic efficacy, reducing cell viability by approximately 75% after laser treatment. These results show that features of gold nanostars, like photothermal properties and anticancer activity, are mostly dependent on their branch morphology, with longer arms providing superior NIR absorption and therapeutic performance for photothermal cancer therapy [30].

### 3.3. MRI-Based Imaging

Zhang et al. developed a triple-negative breast cancer (TNBC)-targeted theranostic nanoplatform as AuNS@MOF-ZD2, by encapsulating a single gold nanostar within a MIL-101-NH_2_(Fe) metal–organic framework with the surface functionalized with the TNBC-specific peptide ZD2. The nanocomposites showed good biocompatibility, efficient capability of T_1_-weighted magnetic resonance (MR) imaging, also it exhibited a stable photothermal performance around 40.5%. In vitro and in vivo studies demonstrated significant MR imaging-guided photothermal therapy upon low-power of laser irradiation of 808 nm. Above that, AuNS@MOF-ZD2 showed high selection targeting toward MDA-MB-231 triple-negative breast cancer cells, while exhibiting minimal affinity for other breast cancer subtypes such as MDA-MB-468, MDA-MB-435, and MCF-7 cells. These results represent the potential of gold nanostar–MOF hybrid nanoplatforms for imaging and photothermal treatment of triple-negative breast cancer with regard to their specific molecular subtype [31].

Hu et al. designed a multifunctional theranostic nanoplatform with folic acid-targeted Fe_3_O_4_@Au core–shell gold nanostars for imaging-guided photothermal cancer therapy. They engineered a magnetic Fe_3_O_4_ core with a plasmonic star-shaped gold shell, supporting complementary MR, CT, and photoacoustic imaging capabilities. Functionalization of the surface with polyethyleneimine and folic acid employed colloidal stability, biocompatibility, and selective targeting on cancer cells with folate receptor overexpression. Near-infrared showed high absorption of the gold nanostar shell and further efficient photothermal tumor ablation. Their model offers a versatile and high-performance multimodal imaging-guided photothermal theranostic platform with potential for various translational applications [32].

### 3.4. Imaging-Related Therapeutic Platforms

Ning et al. conducted a study on mesenchymal stem cells (MSCs) loaded with mesoporous silica-coated gold nanostars (MAuNSs) and indocyanine green as a multifunctional platform for stem cell tracking and PTT in breast cancer. The MAuNSs promoted multimodal imaging, including photoacoustic, fluorescence, and photothermal imaging, enabling real-time monitoring of stem cell distribution in vivo. Three-dimensional photoacoustic imaging demonstrated maximal tumor accumulation of MSCs at 24 h post-injection, providing the optimal function for therapy. In addition, PTT following either intratumoral or intravenous administration effectively inhibited tumor growth and reduced recurrence. These results highlight the potential of gold nanostar-based stem cell platforms for image-guided cancer therapy and real-time tracking of therapeutic cells [33].

Hyojin Lee et al. introduced Anti-HER2 aptamer-functionalized gold nanostars (HApt-AuNS) to enhance targeted therapy against HER2-positive breast cancer cells. Following specific binding to HER2 receptors, the nanoconstructs were internalized through HER2-mediated endocytosis and progressively trafficked from early endosomes to lysosomes. Within the acidic lysosomal environment, HER2 underwent enzymatic degradation, leading to increased lysosomal activity, apoptosis, cell-cycle arrest in the G0/G1 phase, and inhibition of cancer cell proliferation. The accumulation of HApt-AuNS complexes in lysosomes was positively correlated with HER2 degradation and therapeutic efficacy that, based on gold nanostars, can exploit lysosomal trafficking and receptor degradation pathways to enhance the anticancer activity of aptamer-based therapeutics, providing a promising strategy for targeted treatment of HER2-positive breast cancer [34].

**Figure 4:**
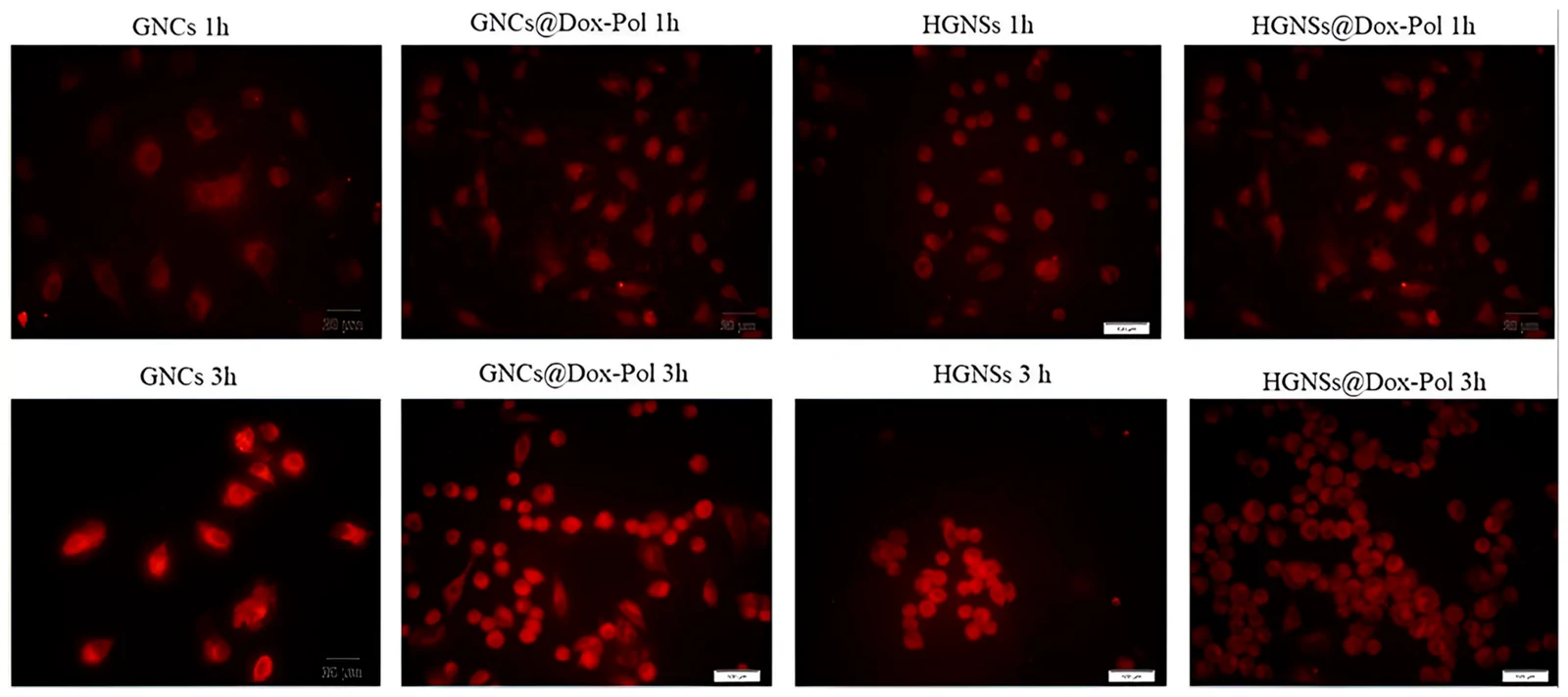
Fluorescent microscopy images showing cellular uptake of rhodamine B-labeled AuNSs and AuNSs@Dox-Pol in MCF-7 breast cancer cells after 1 and 3 h of incubation. Reprinted from ref. [35]. Copyright 2021, Cancer Nanotechnology. This article is an open-access article distributed under the terms and conditions of the Creative Commons Attribution (CC BY) license.

Pakravan et al. showed the comparative effects of thermo-pH-responsive polymer-coated gold nanocages and hollow nanostars on chemo-photothermal therapy of breast cancer cells. Doxorubicin-loaded hollow gold nanostars (HAuNSs@Dox-Pol) were developed as thermo- and pH-responsive nanoplatforms for combined chemotherapy and photothermal therapy of breast cancer. The nanoparticles exhibited rapid uptake by MCF-7 breast cancer cells within 3 h (Figure 4) and demonstrated efficient photothermal conversion under near-infrared irradiation, generating temperature increases of 23–27 °C. Compared with gold nanocages, hollow gold nanostars showed superior photothermal performance and enhanced therapeutic efficacy. Notably, the combined chemo-photothermal treatment induced up to 90% cell mortality and 97% apoptosis in MCF-7 cells, highlighting the potential of doxorubicin-loaded hollow gold nanostars as effective agents for synergistic breast cancer therapy [35].

Tan et al. developed a multifunctional gold nanostar-based nanoplatform for combination targeted and image-guided therapy of castration-resistant prostate cancer. The GNS@IR820/DTX-CD133 nanoprobe was evaluated in vitro and in vivo to assess the performance of photothermal, photodynamic, and chemotherapeutic upon near-infrared irradiation. In vitro results showed efficient light-to-heat conversion, enhanced singlet oxygen generation, NIR-triggered drug release, and strong affinity toward prostate cancer cells (PC3), leading to effective tumor cell killing. In vivo studies on mice confirmed high tumor-targeting efficiency and significant antitumor efficacy, monitored by NIR fluorescence and photoacoustic imaging. Thus, this PEGylated, CD133-targeted nanoplatform enables synergistic PTT/PDT/CT with high therapeutic results, offering its future potential for advanced prostate cancer treatment in precision nanomedicine [36].

Sato et al. suggested a simple and effective strategy for constructing extracellular vesicle–gold nanostar (EV-GNS) hybrid nanocomplexes for photothermal cancer therapy. By exploiting electrostatic reactions between negatively charged extracellular vesicles and cationic PEG-modified gold nanostars, they generated stable EV-GNS nanocomplexes while preserving key biological characteristics of EVs. The hybrid system introduced both the intrinsic biological functionality of EVs with the efficient photothermal conversion properties of gold nanostars, and improved both photothermal performance and laser-responsive cytotoxicity in vitro. These findings suggest that EV-based gold nanostar nanocomplexes represent a promising delivery platform for gold-mediated photothermal therapy [37].

The biological interaction and cellular application of gold nanostars show specific chemical reactions and shape-dependent effects on cancer cell behavior. Carolina Carrillo-Carrión et al. introduced a microporous plasmonic nanoreactor capable of performing photothermal-driven chemical reactions inside living cells. Through integrating a gold nanostar core within a zeolitic imidazole framework-8 (ZIF-8)-based metal–organic framework shell, they created a system that combined efficient near-infrared photothermal conversion with controlled molecular diffusion. Following light activation, the nanoreactor produced localized heating sufficient to induce high-energy intramolecular cyclization reactions under physiological conditions. Also, yielding fluorescent products generate direct visualization of intracellular chemistry. This strategy can be considered a powerful approach for spatiotemporally controlled chemical transformations in biological environments and demonstrates the potential of plasmonic nanoreactors for applications like intracellular synthesis and prodrug activation [38].

**Figure 5:**
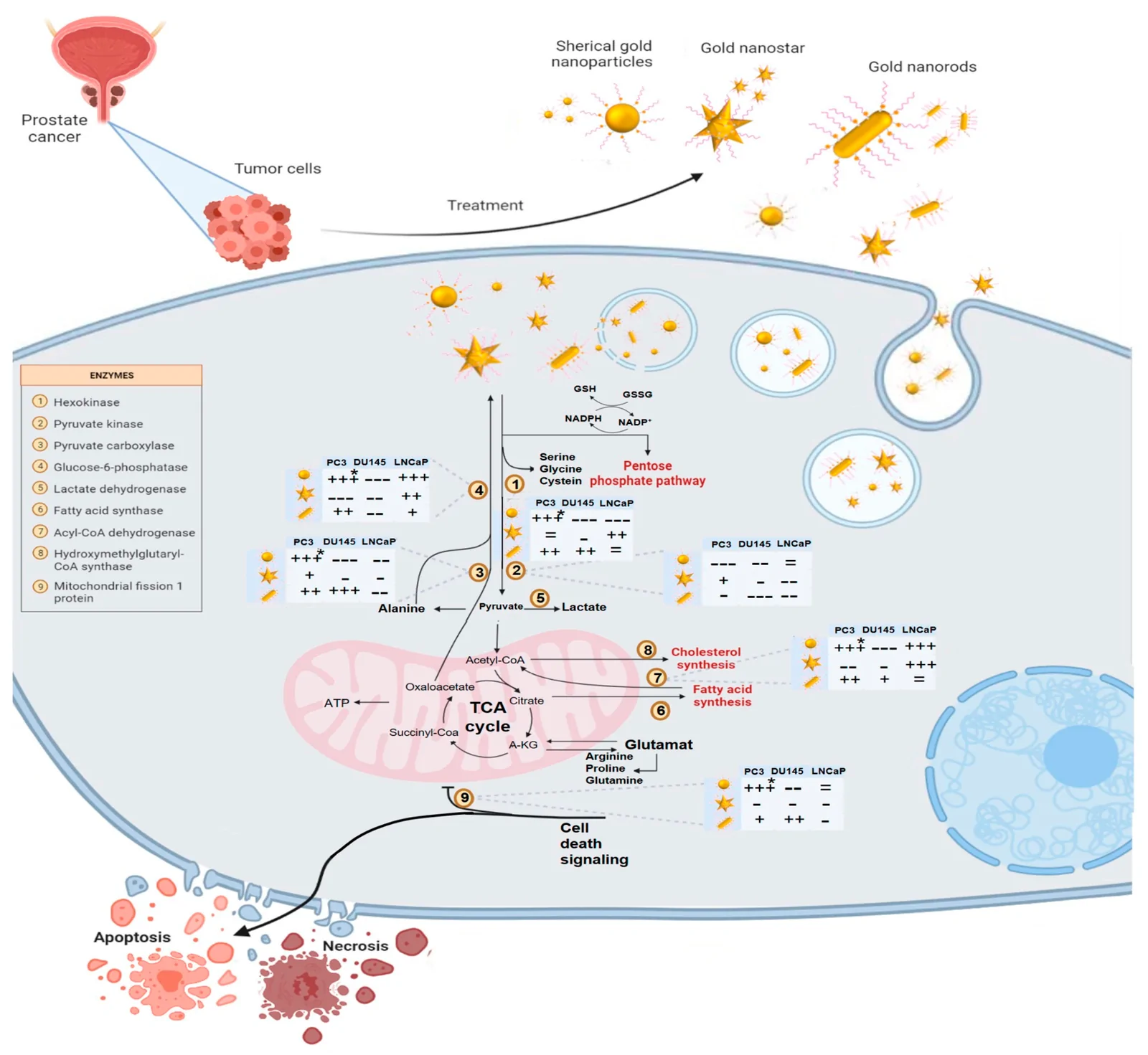
Effect of different types of gold nanoparticles (AuNPs) on metabolic pathways in prostate cancer cells following cellular internalization, with particular emphasis on glycolysis, gluconeogenesis, and β-oxidation. Prostate cancer cells display enhanced lipid metabolism, which results in citrate synthesis at the tricarboxylic acid (TCA) cycle serves not only as an energy-related metabolite but also as a precursor for the synthesis of other biomolecules. In addition, specific gluconeogenic reactions may be activated to maintain normal glucose levels which can then be used for anabolic purposes. The expression levels of hexokinase-2, pyruvate kinase, glucose-6-phosphatase, pyruvate carboxylase, acyl-CoA dehydrogenase, and mitochondrial fission protein 1 were measured using RT-qPCR. The effect of AuNPs-PEG in the expression of metabolic enzymes represented by using +, − or = symbols (+++ > ++ > + and −−− > −− > −). (*) *p* < 0.05. Reprinted from ref. [39]. Copyright 2023, Cells. This article is an open-access article distributed under the terms and conditions of the Creative Commons Attribution (CC BY-NC-ND 4.0) license.

Soares et al. evaluated three different PEGylated gold nanoparticle morphologies such as spherical (AuNPsp), star-shaped (AuNPst), and rod-shaped (AuNPr) on prostate cancer cell behavior (Figure 5). They used PC3, DU145, and LNCaP cell lines, and all nanoparticle structures were internalized by the cells successfully; however, their biological responses were strongly shape-dependent. The cellular metabolic activity in prostate cancer cell lines PC3 and DU145 followed the order AuNPsp-PEG, AuNPst-PEG, AuNPr-PEG, showing shape-dependent metabolic modulation. AuNPr-PEG induced the highest reduction in cellular proliferation in PC3 and DU145 cells, whereas AuNPst-PEG exhibited the lowest toxicity in LNCaP cells. In LNCaP cells, proliferation was slightly stimulated at different concentrations ranging from 0.001–0.1 mM, while a significant decrease in proliferation was observed only at 1 mM AuNPr-PEG. Further analysis in reactive oxygen species production and RT-qPCR indicated that the shape of nanoparticles influenced cellular metabolic pathways. They highlighted that the biological activity of PEGylated gold nanoparticles is highly dependent on particle shape, emphasizing the importance of morphology optimization for nanomedicine and cancer-related applications [39].

### 3.5. Applications in Biosensing—Cytosensing for Cancer Detection

Amin Zhang et al. introduced a sensitive electrochemical cytosensor based on gold nanostar-decorated graphene oxide nanocomposites functionalized with an rBSA-FA bio-complex for gastric cancer cell detection. By integrating the high conductivity of graphene oxide and gold nanostars with plasmonic and surface properties, they generated a constructed sensing platform that achieved both improved electrochemical performance and good biocompatibility. The incorporation of folic acid through rBSA-FA also enhanced stability and selective recognition of gastric cancer cell lines (MGC-803). The cytosensor demonstrated a low-detection limit and a broad linear range, which highlights its strong potential for application in clinical cancer diagnosis and sensitive cellular analysis [40]. In another study Wang et al. showed a highly sensitive and specific biosensing strategy based on a TiO_2_-modified interdigitated electrode platform for the early detection of head and neck cancer. A clinically relevant serum biomarker correlated with tumor burden as squamous cell carcinoma antigen was selectively detected using antibody-functionalized sensing surfaces. Conjugation of the antibody with gold nanostars resulted in a substantial enhancement in sensitivity and by several orders of magnitude, achieving femtomolar-level detection and outperforming conventional substrates. The demonstrated specificity and sensitivity emphasized the potential of gold nanostar-assisted IDE-TiO_2_ sensors as favorable diagnostic tools for early-stage head and neck cancer detection [41].

Also, Chenaghlou et al. developed a biosensor based on gold nanostar-enhanced electrochemiluminescence (ECL) for the sensitive detection of the cancer stem cell biomarker CD133 peptide. In this platform, graphitic carbon nitride (g-CN) nanosheets were functionalized with gold nanostars (AuNSs) to generate a strong localized surface plasmon resonance on multiple branched tips of the nanostars. The AuNSs@g-CN nanocomposite in comparison with pure g-CN nanosheets exhibited significant stable cathodic ECL emission, with signal intensities more than 30%, superior to those obtained using spherical gold nanoparticles. Excellent reproducibility of biosensors demonstrated a relative standard deviation of less than 4% over 10 consecutive scanning cycles. Anti-CD133-functionalized electrodes decreased ECL signal linearly, also with CD133 peptide in different concentrations rang from 0.05 to 100 ng mL^−1^, achieving a limit of detection (LOD) of 0.257 ng mL^−1^. Furthermore, successful detection of CD133 in spiked serum samples confirmed the practical applicability of the platform, demonstrating that gold nanostars can significantly enhance ECL performance through plasmonic effects, enabling highly sensitive and reliable detection of cancer stem cell biomarkers for potential diagnostic applications [42].

## 4. Nanostars Used for Therapy

Studies of gold nanostar-based therapies are provided in summarized form in Table 2 as an overview and are subsequently discussed in the following subsections for more detailed consideration of each study.

### 4.1. Phototherapy: Photothermal/Photodynamic/Photoimmunotherapy

Conventional brain tumor therapies rely on surgical resection followed by chemotherapy or radiotherapy, approaches that are invasive and associated with significant neurological and systemic side effects. Hamed Aram et al. introduced an integrated nanomedicine–bioelectronics brain machine interface enabling minimally invasive, on-demand tumor treatment. Gold nanostars with near-infrared plasmonic properties allow selective photothermal ablation of deep brain tumors in freely behaving mice. Surface functionalization promotes tumor-specific diffusion and localized heating, while wireless control enables precise adjustment of therapeutic parameters for different brain regions which allowed remote control of irradiation parameters, including light wavelength, power, and photothermal intensity, providing a versatile strategy for targeted treatment of brain tumors without the need for open-skull surgery or the systemic toxicities associated with conventional chemotherapy and radiotherapy [43].

Liu et al. conducted a new synergistic immuno-photothermal nanotherapy (SYMPHONY) which combined plasmonic gold nanostar-mediated photothermal therapy with immune checkpoint blockade. A murine flank tumor model based on the CT-2A glioma cell line used. SYMPHONY produced durable therapeutic responses, including long-term survival and resistance to tumor rechallenge, showing immunologic memory induction. This work highlighted the first preclinical investigation of gold nanostar-based photoimmunotherapy for GBM and suggests its potential as an effective immune-activating treatment strategy for aggressive brain tumors [44].

Zhu et al. suggested that gold nanomaterials and carbon nanotubes are promising photothermal agents for cancer therapy. They reported a surfactant-free gold nanostar multiwalled carbon nanotube hybrid synthesized via controlled two-step reduction. The hybrid structure exhibited high biocompatibility and significant photothermal conversion with gold nanostars and nanospheres under 808 nm irradiation. At low concentrations, it demonstrated marked cancer cell ablation improvement, attributed to plasmonic absorption and synergistic coupling effects, highlighting its potential as an accurate photothermal therapeutic platform [45].

Zhao et al. offered a multifunctional gold nanostar platform that integrates intrinsic dual nanozyme activity with photothermal therapy for enhanced cancer treatment. Synthesized using a simple and environmentally friendly one-step method, the gold nanostars simultaneously catalyzedd glucose oxidation and hydrogen peroxide decomposition, generating cytotoxic hydroxyl radicals to amplify cascade catalytic therapy. Also, the photothermal effect boosted catalytic efficiency, making effective synergy between catalytic and thermal treatment modalities. Photoacoustic imaging guidance helped nanostars achieve highly efficient tumor ablation with a particularly high antitumor rate. This study described a practical strategy for using single component gold nanoparticles to overcome limitations in photothermal therapy and advance synergistic nanomedicine-based cancer treatments [46].

Ramirez Henao et al. illustrated a novel gold nanostar-based hybrid nanocomposite synthesis with reduced bisphosphonate-functionalized polyoxometalate as a dual-function reducing and capping agent. The produced AuNS@POM integrates alendronate ligands, redox-active molybdenum centers, and plasmonic gold within a single platform. It showed excellent stability under biological conditions. The oxidation of Mo(V) during nanostar formation improves the intrinsic antitumoral activity of the polyoxometalate, while near-infrared irradiation activated the photothermal effect of the gold nanostars. This combination of chemo-photothermal action leads to pronounced killing of cancer cells in both glioblastoma and liver cancer models. All results observed a promising dual therapeutic agent of AuNS@POM for near-infrared-triggered cancer treatment [47].

Li et al. developed a multifunctional theranostic nanosystem that integrates natural product-derived photosensitizers with plasmonic photothermal agents for enhanced cancer therapy. By using aggregation-induced emission properties, the berberine dimer BD3 promoted efficient singlet oxygen generation for photodynamic therapy but gold nanostars provided strong photothermal conversion. Functionalization of hyaluronic acid induced CD44 targeted delivery to breast cancer cells, and their results showed synergistically enhanced therapeutic outcomes. The strategy of combining PDT/PTT to produce strong apoptotic and necrotic responses in cancer cells also exhibited effective tumor suppression in vivo. This work introduced a promising approach for involving natural product derivatives with photothermal nanomaterials to advance cancer therapies [48].

**Figure 6:**
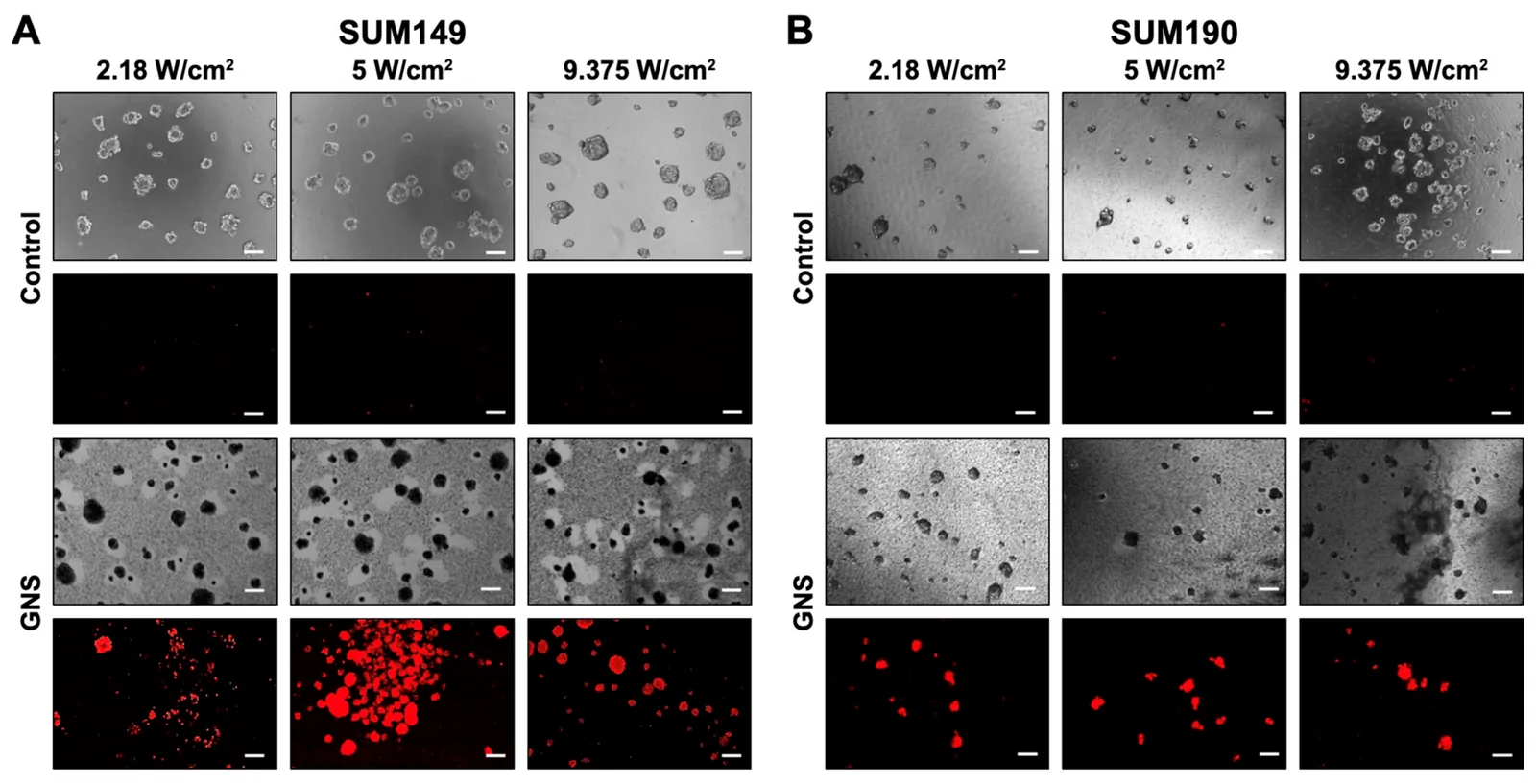
Photothermal therapy of SUM149 (**A**) and SUM190 (**B**) tumor emboli. Tumor emboli were allowed to mature for 96 h, followed by incubation with 0.15 nM gold nanostars (AuNSs) for 24 h. The emboli were subsequently irradiated using an 808 nm continuous-wave laser at different power densities. Following treatment, samples were stained with propidium iodide to assess treatment-induced cell death. GNS-treated emboli exhibited extensive cell death after laser irradiation, whereas control emboli exposed to identical irradiation conditions showed minimal or no detectable cell death. Representative phase-contrast images were acquired after treatment. The empty regions observed in GNS-treated emboli are attributed to emboli displacement within the wells during photothermal irradiation. Under phase-contrast microscopy, GNS-labeled emboli appear darker because of intracellular nanoparticle accumulation, in contrast to the unlabeled control emboli. Scale bars = 100 μm. Reprinted from ref. [49] under CC BY license.

Crawford et al. evaluated gold nanostars as multifunctional imaging and photothermal agents for inflammatory breast cancer (IBC) using 3D tumor emboli models generated from SUM149 and SUM190 cell lines (Figure 6). The nanostars showed high cellular uptake efficacy and enhanced penetration into tumor embolic cores regardless of receptor status or drug resistance. Due to their strong plasmonic and photothermal properties, AuNSs could improve multiphoton imaging and effective photothermal ablation of tumor emboli after irradiation. These findings suggested gold nanostars’ potential for imaging-guided photothermal treatment of aggressive inflammatory breast cancer [49].

Li et al. introduced chlorin e6-conjugated and polydopamine-coated gold nanostars (AuNSs@PDA-Ce6) used as multifunctional phototheranostic nanoprobes for photoacoustic imaging and combined photothermal/photodynamic therapy of metastatic breast cancer. The polydopamine coating enhanced photothermal conversion efficiency and photoacoustic imaging performance compared with bare gold nanostars, while Ce6 conjugation enabled efficient singlet oxygen generation for photodynamic therapy. Under near-infrared laser irradiation, the combined PTT/PDT treatment significantly inhibited the growth of 4T1 breast tumors and reduced lung metastasis. These findings demonstrate the potential of AuNSs@PDA-Ce6 as effective nanoplatforms for precision imaging-guided therapy and metastasis suppression in breast cancer [50].

Chunhui Wu et al. reported a multifunctional graphene–gold nanostar-based phototheranostic platform for integrated cancer imaging and therapy. Combining the strong near-infrared absorbance of graphene and gold nanostars with photosensitizer assembly produced a nanocomposite with efficient photothermal conversion achievement and potent photodynamic activity. Spectral matching with Chlorin e6 (Ce6) promoted activation of photodynamic and photothermal effects simultaneously with using a single laser wavelength, leading to complete tumor eradication in vivo. The cellular uptake enhancement and following disruption of lysosomes and mitochondria contributed to the synergistic anticancer mechanism. In total, the excellent biocompatibility and minimal off-target toxicity of this nanoplatform demonstrated its potential as a reliable system for photo-controlled cancer theranostic applications [51].

Zhang et al. developed a multifunctional gold nanostar-based nanoprobe for CT imaging-guided synergistic photothermal and photodynamic cancer therapy. The gold nanostars platform produced for photothermal therapy incorporated an Ce6-loaded mesoporous silica shell for photodynamic therapy to generate catalase-mediated alleviation of tumor hypoxia by converting hydrogen peroxide into oxygen and c(RGDyK)-modified phospholipids for tumor targeting. The nanoprobe showed high stability, photothermal conversion efficientcy around ~28%, and a photo-controlled (on–off) release of Ce6 triggered through photothermal stimulation. In vivo observation showed strong CT contrast at tumor sites 72 h after injection, and combined catalase-enhanced PDT/PTT therapy indicated greater antitumor efficacy compared to monotherapy alone. These findings highlight the potential of targeted gold nanostars for hypoxia-responsive multimodal cancer theranostics [52].

Malik et al. highlighted the key role of branch morphology in determining the biological performance of aptamer-functionalized gold nanostars for targeted cancer therapy. By considerably controlling the synthesis conditions, longer and sharper AuNS branches can effectively mitigate protein corona interference with preserving receptor-specific interactions in serum-rich environments. As a result, AuNSs with optimized branch morphology and HER2 targeting showed improvement on cytotoxic effects in HER2-overexpressing cancer cells compared to spheroidal nanoparticles or nanostars with shorter branches. All results provided important design principles for improving receptor-mediated targeting and therapeutic efficacy of gold nanostar-based nanomedicines in complex biological systems [53].

Mohanna Etemad et al. provided a computational framework for optimizing gold nanostar-mediated photothermal therapy using finite element modeling. The model captures temperature evolution within tumors and adjacent tissues under near-infrared laser irradiation, simulating heat generation and transfer based on the bioheat equation. The results demonstrated that considering laser parameters and heating strategies is essential to avoid surface overheating while ensuring effective tumor destruction. Especially, the study established quantitative guidelines for treatment time, laser intensity, irradiation radius, and temperature thresholds, suggesting valuable insights for the possible safe and efficient design of gold nanostar-based photothermal cancer therapies [54].

Soleimany et al. developed a nanohybrid composed of thiolated chitosan-coated gold nanostars (AuNS-TCS) and riboflavin-conjugated N, S-doped graphene quantum dots (Rf-N, S-GQDs) for single-laser-triggered combined photodynamic and photothermal therapy (PDT/PTT). The gold nanostars acted as photothermal agents, while the graphene quantum dots served as two-photon photosensitizers, enabling synergistic therapy under low-power pulsed laser irradiation. The thiolated chitosan coating improved nanoparticle stability and facilitated electrostatic binding of the quantum dots. Due to plasmon-enhanced singlet oxygen generation, the nanohybrid produced enhanced extracellular and intracellular reactive oxygen species, resulting in significantly improved therapeutic efficacy in both 2D cancer cells and 3D multicellular tumor spheroids compared with individual PDT or PTT treatments. These findings demonstrate the potential of gold nanostar-based plasmonic hybrids for efficient single-laser combined cancer phototherapy [55].

Wang et al. developed PEGylated mixed-charge gold nanostars functionalized with amine- and carboxyl-terminated polyethylene glycol as pH-responsive photothermal agents for tumor-selective therapy. The nanostars exhibited reversible changes in cellular affinity and therapeutic activity in response to the extracellular pH difference between normal tissues (pH 7.4) and tumors (pH 6.4), while maintaining good colloidal stability and photothermal performance. Among the tested formulations, GNS-N/C 4 showed strong cellular uptake and enhanced photothermal efficacy under acidic tumor-like conditions, but minimal interaction and nearly no toxicity at physiological pH. In vivo studies further demonstrated increased tumor accumulation and improved photothermal therapeutic efficacy compared with pH-insensitive nanostars. These findings highlight the potential of pH-responsive gold nanostars for selective and safer photothermal cancer therapy [56].

Kim et al. conducted a study in which red blood cell and platelet membrane-coated gold nanostars loaded with curcumin (R/P-cGNS) were created as biomimetic nanoplatforms for targeted melanoma therapy. The platelet membrane enhanced targeting toward cancer cells, with the result that red blood cell membrane coating enabled immune evasion by reducing macrophage clearance. In addition, curcumin provided complementary anticancer and anti-inflammatory effects. The nanostars generated controlled drug release under near-infrared irradiation and enhanced anticancer efficacy together with immunomodulatory activity on macrophages. These outcomes illustrated that membrane-coated gold nanostars represent a promising hybrid drug delivery system capable of improving tumor targeting, photothermal therapy, and immune compatibility for cancer treatment [57].

Fangfang Xia et al. developed a multifunctional gold nanostar-based nanotheranostic platform for targeted lung cancer imaging and therapy. By integrating IR-780 iodide and MMP2-responsive polypeptides onto a bovine serum albumin-coated gold nanostar scaffold, the nanoprobe showed dual-modal photoacoustic and near-infrared fluorescence imaging alongside synergistic photothermal and photodynamic therapy. The MMP2 polypeptide increased tumor targeting, while the combined action of IR-780 and gold nanostars generated efficient heat and reactive oxygen species generation following a single light source. The engineered nanoprobes indicated strong antitumor efficacy with substantial suppression of tumor and good biocompatibility, highlighting their role as integrated diagnostic and therapeutic agents for personalized cancer therapy [58].

Li et al. conducted research on integrin αvβ3-targeted polydopamine-coated gold nanostars for photothermal ablation therapy of hepatocellular carcinoma. They developed targeted RGDpeptide-conjugated polydopamine-coated gold nanostars (Au@PDA-RGD NPs) as photothermal agents for hepatocellular carcinoma (HCC). The gold nanostars were coated with a polydopamine shell also functionalized with polyethylene glycol and RGD peptides with the aim of enhancing biocompatibility and targeting αVβ3 integrin-overexpressing HepG2 liver cancer cells. The near-infrared absorption at 822 nm exhibited a strong effect in nanoparticles, enabling appropriate photothermal therapy. In vitro testing resulted in effective targeted killing of HepG2 cells, while mechanistic investigations revealed that the treatment caused cancer cell death through dysfunction of mitochondrial lysosomal and autophagy-related pathways. Additionally, in vivo experiments on mice confirmed remarkable tumor suppression with minimal systemic toxicity, suggesting the potential of Au@PDA-RGD nanostars as both targeted and effective photothermal platform for hepatocellular carcinoma therapy [59].

Bladder cancer frequently presents at advanced or metastatic stages, limiting the effectiveness of conventional therapies. So, Ericka Chorniak et al. developed a novel photoimmunotherapy that integrates gold nanostar-mediated photothermal therapy with immune checkpoint inhibition for bladder cancer treatment. In murine models, the combined method demonstrated higher therapeutic accuracy in comparison with each monotherapy alone. Using intravital optical imaging in a dorsal skinfold window chamber model made dynamically tracked immune responses in distant tumors. CX3CR1-GFP-expressing mice promoted visualization of monocytes, natural killer cells, and dendritic cells, and metastatic lesions showed enhanced immune cell infiltration. The results demonstrate that photoimmunotherapy generates systemic antitumor immune responses and suggests intravital optical imaging as a powerful tool for mechanistic evaluation of cancer immunotherapies [60].

### 4.2. Drug Delivery and Chemo-Photothermal Therapy

Liang et al. presented a cell–drug integrated nanoplatform designed to maximize tumor accumulation and therapeutic efficacy through immune cell-mediated delivery. By conjugating gold nanostars with indocyanine green and trastuzumab, and loading them into tumor-tropic CIK cells, they achieved enhanced intratumoral targeting and homogeneous distribution, as validated by tri-modal imaging. The platform enables synergistic photothermal and photodynamic therapy under near-infrared irradiation while simultaneously activating antitumor immune responses through CIK cells. The specific interaction between trastuzumab and HER-2-positive tumor cells further improve therapeutic precision. Overall, this work demonstrates a promising strategy for combining multimodal imaging with multimodal therapy and highlights the potential of immune cell-assisted nanomedicine delivery in cancer theranostics [61].

Xu et al. developed a cucurbit [7] uril (CB [7]) stabilized gold nanostar platform for NIR-triggered drug release and synergistic chemo-photothermal cancer therapy. In this system, the anticancer drug camptothecin (CPT) was encapsulated through host–guest interactions with CB [7], which also improved the colloidal stability of the gold nanostars. Upon NIR irradiation, controlled drug release was triggered together with the photothermal effect of the nanostars, enabling combined chemotherapy and photothermal therapy. This multifunctional supramolecular platform demonstrates the potential of gold nanostars for remotely controlled drug delivery and synergistic cancer treatment [62].

Yingke Hou et al. developed a multifunctional gold nanostar-hierarchical nanoplatform for breast cancer therapy by synergistic radiosensitization and photothermal modulation. Using glutathione-responsive camptothecin prodrugs with fluorescence tracking and hyaluronic acid-mediated CD44 targeting indicated precise drug activation and real-time visualization within tumor cells. The high atomic number of the gold nanostars improved deposition of radiation energy, while mild photothermal therapy alleviated tumor hypoxia and all procedure amplifying radiosensitization effects. This combination of mechanisms achieved strong immunogenic cell death and enhanced therapeutic outcomes. The model offers an effective strategy for constructing compact, multifunctional nanostructures for theranostic use that modulate the tumor microenvironment in breast cancer treatment [63].

Liu et al. proposed a calcium carbonate-encapsulated gold nanostar platform loaded with indocyanine green (GNS@CaCO3/ICG) as a pH-responsive combined photodynamic and photothermal therapeutic cancer. In this system, the gold nanostars provide photothermal activity, while the indocyanine green improves both photodynamic therapy and near-infrared fluorescence imaging. The platform is both a drug reservoir and a pH-responsive trigger, enacted by the calcium carbonate shell, enabling tumor-specific drug release but protecting ICG from rapid blood clearance. Under near-infrared irradiation, the nanoplatform resulted in higher antitumor efficacy compared with individual PDT or PTT therapies alone. Additionally, the nanoparticles enabled tumor-targeted fluorescence, photoacoustic, and two-photon imaging, allowing monitoring of biodistribution, drug release, and tumor accumulation. This multifunctional gold nanostar-based system suggests an effective method for image-guided synergistic cancer therapy and controlled drug delivery [64].

Wei et al. developed RGD-modified dendrimer-stabilized gold nanostars (RGD-Au DSNSs) for targeted CT imaging, thermal imaging, photothermal therapy, and VEGF siRNA delivery. The nanostar systems targeted αvβ3 integrin-overexpressing cancer cells selectively and showed increased anticancer efficacy under near-infrared irradiation, reducing cell viability to approximately 20.2% via both photothermal and gene therapy. Furthermore, in vivo testing confirmed effective tumor imaging and synergistic therapeutic performance, highlighting the potential of multifunctional gold nanostars for imaging-guided cancer therapy [65].

Peilin Huang et al. introduced β-cyclodextrin-functionalized mesoporous silica and magnetic mesoporous silica nanostars as multifunctional drug delivery systems for curcumin. Through a bifunctionalization strategy, β-cyclodextrin acted as a hydrophilic carrier and pH-responsive gate, improving curcumin solubility, bioavailability, and controlled release behavior. The nanoplatforms demonstrated high biocompatibility and significantly superior anticancer activity against SK-HEP1 and HepG2 cells, in comparison with free curcumin. PH-responsive and magnetically controlled drug release was shown in in vitro studies, while confocal microscopy confirmed an increase in intracellular uptake and release of curcumin. The potential of functionalized mesoporous silica nanostars highlights its role as an effective platform for controlled delivery of hydrophobic anticancer drugs [66].

Enea et al. evaluated two different shapes of gold nanoparticles, citrate- and MUA-capped spherical and star-shaped, for cytotoxicity, cellular uptake, and intestinal permeability in both in vitro and in vivo tests using Caco-2 colon adenocarcinoma cells and Wistar rats. Smaller citrate-capped nanoparticles (~15 nm) displayed higher toxicity than larger ones or MUA-coated nanoparticles, while the presence of 1% fetal bovine serum improved nanoparticle stability and reduced cytotoxicity significantly. Cellular uptake was enhanced in serum-supplemented media, especially for 60 nm nanoparticles, although none of the tested nanoparticles crossed the intestinal barrier in vitro. More than that, in vivo experiments showed low systemic absorption of orally administered 60 nm gold nanospheres and nanostars, with most nanoparticles removed through feces. As a result, gold nanostars possess good biocompatibility and potential for intestinal drug delivery or diagnostic applications, but more functionalization may be required to improve bioavailability [67].

Navarro et al. provided a comprehensive analysis of PEGylated gold nanostars and bipyramidal-like gold nanoparticles as plasmonic platforms for biological applications. They used a correlating ensemble of optical measurements with single-particle spectroscopy and transmission electron microscopy and established a clear relationship between nanoparticle geometry and plasmonic response. Biocompatible features of gold nanostars with PEG coatings improved stable dispersion and facilitated interaction with biological systems. Cellular studies demonstrated efficient uptake and intracellular localization of both nanostructures in melanoma cells, which also demonstrates their potential for bioimaging and biosensing applications where sensitivity to the local environment is critical [68].

### 4.3. Radiotheranostic Applications

Liu et al. conducted research on ^211^At -labeled gold nanostars as a promising platform for targeted alpha-particle therapy (TAT). The nanostars achieved efficient radiolabeling around 100% within 1 min and indicated excellent stability, retaining ^211^At activity at more than 99% after 24 h in serum. In vivo biodistribution studies showed minimal deastatination, with low uptake in the thyroid (0.44–0.64% ID) and stomach (0.21–0.49% ID), indicating strong radiolabel stability. In a U87MG human glioma xenograft mouse model, intratumoral administration of ^211^At -AuNSs significantly suppressed tumor growth. These findings highlight gold nanostars as highly stable and efficient carriers for ^211^At-based targeted alpha therapy, with strong potential for future cancer theranostic applications [69].

In a study by Yang Liu and colleagues, a novel gold nanoprobe, designated 124I-GNS, was engineered for the sensitive detection and high-resolution spatiotemporal tracking of brain tumors. Employing optical imaging and transmission electron microscopy (TEM) in a murine orthotopic glioma model, the authors demonstrated that systemically administered 124I-AuNSs efficiently extravasated from the tumor vasculature, penetrated the interstitial matrix, and underwent cellular internalization via endocytosis. The probe exhibited a high tumor-to-normal (T/N) uptake ratio and remarkable sensitivity, facilitating the detection of lesions measuring as small as 0.5 mm, a resolution that exceeded the capabilities of currently available non-invasive imaging modalities. Concurrent in vivo toxicity assessments over a six-month period revealed no significant physiological adverse effects following systemic administration. Collectively, these findings establish the 124I-GNS nanoprobe as a biocompatible and ultrasensitive imaging agent, underscoring its considerable translational promise for the clinical management of brain tumors [70].

Cutshaw et al. introduced multimodal gold nanostars (MGNs) functionalized with Raman reporters, immune-targeting antibodies, and NOTA-chelated ^64^Cu radiolabels as dual-imaging probes for real-time monitoring of immune responses during cancer immunotherapy. By combining the whole-body sensitivity of immunoPET with the high spatial resolution and multiplexing capability of SERS, the MGNs enabled simultaneous in vivo detection of CD8^+^ T cells and NKp46^+^ natural killer (NK) cells within the tumor microenvironment. The nanoprobes were evaluated in CT26 immunologically “hot” murine tumors, which responded to combination immunotherapy with anti-PD-L1 + anti-CD47, and 4T1 immunologically “cold” tumors, which showed poor therapeutic response. The MGNs successfully tracked immune-cell recruitment and accurately predicted treatment outcomes, while imaging findings were validated using immunofluorescence, ELISA, and flow cytometry. These results showed that gold nanostar-based multimodal imaging made a powerful platform for noninvasive monitoring of immune dynamics and efficient early prediction of immunotherapy, suggesting valuable guidance for personalized cancer treatment decisions [71].

**Figure 7:**
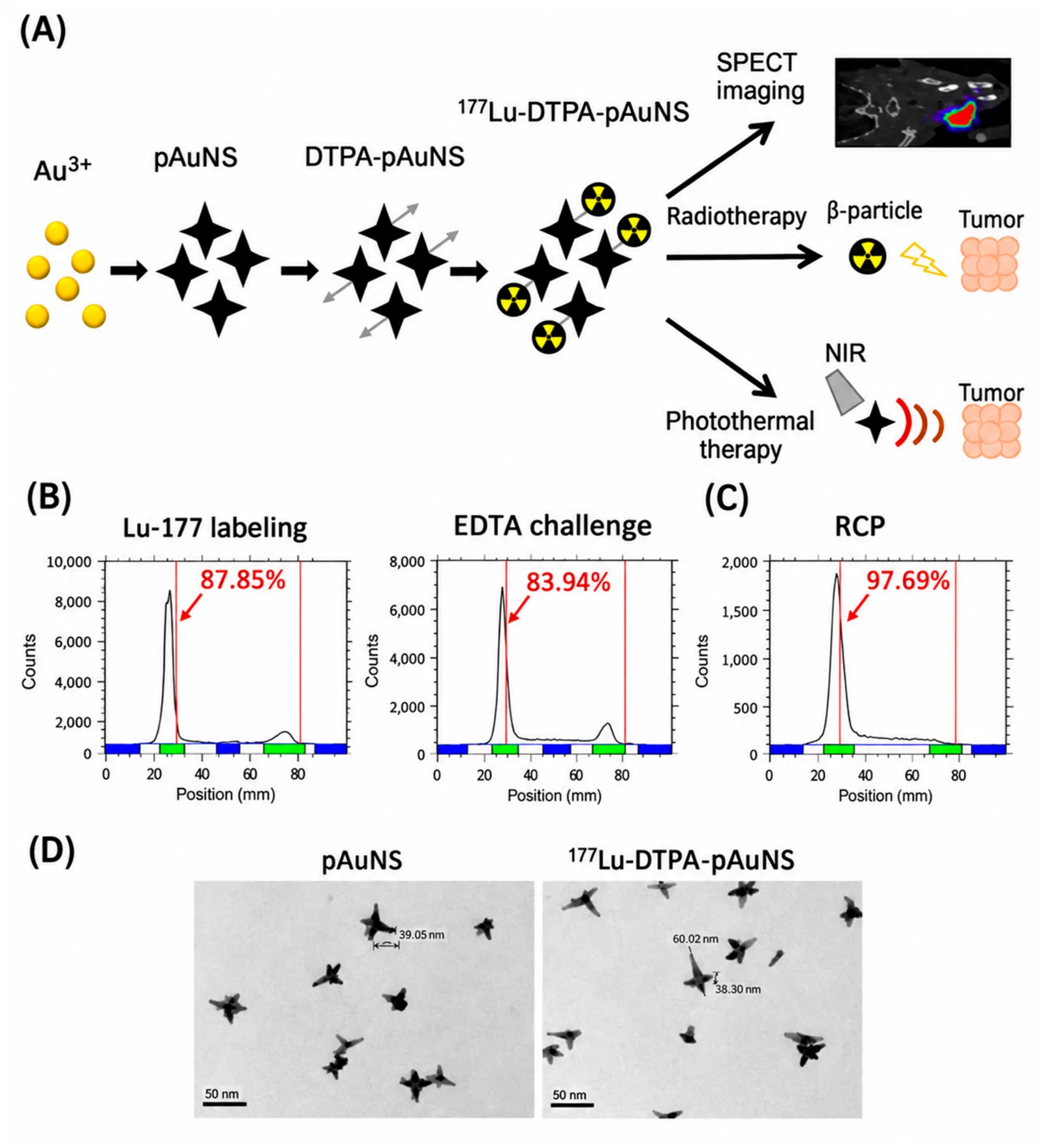
Synthesis and radiolabeling of ^177^Lu-labeled PEGylated gold nanostars (^177^Lu-DTPA-pAuNS). (**A**) Schematic overview of the preparation of ^177^Lu-DTPA-pAuNS and the proposed theranostic applications. (**B**,**C**) RadioTLC analysis demonstrating the labeling efficiency following Lu-177 labeling, EDTA stability test and centrifugation. (**D**) Transmission electron microscopy (TEM) images of pAuNS and ^177^Lu-DTPA-pAuNS. Reprinted from ref. [72]. Copyright 2021, Pharmaceutics. Under CC BY license.

Lin et al. aimed to develop a multifunctional nanoplatform for brachytherapy of head and neck squamous cell carcinoma (HNSCC) using ^177^Lu-labeled PEGylated gold nanostars (^177^Lu-DTPA-pAuNS) (Figure 7). In an orthotopic HNSCC model, the nanostars showed prolonged tumor retention for up to 15 days, whereas free ^177^Lu-DTPA was undetectable after 24 h. The ^177^Lu-DTPA-pAuNS significantly suppressed tumor growth, and the therapeutic effect was further enhanced when combined with photothermal therapy. These findings demonstrate the potential of radiolabeled gold nanostars as effective theranostic platforms for image-guided radiotherapy and combined cancer treatment [72].

Chen et al. demonstrated the potential of PEGylated gold nanostars as effective agents for image-guided photothermal therapy. Synthesized via a simple HEPES reduction method, the nanostars display high photothermal conversion efficiency and stability both in vitro and in vivo. By employing ^111^In-DTPA-labeled nanostars as a radioactive surrogate, the authors successfully achieved noninvasive tracking of gold nanostar biodistribution using microSPECT/CT imaging. The strong correlation between radioactive signal and gold content confirms the reliability of this approach for in vivo monitoring. Therapeutic evaluation in a SKOV-3 xenograft model revealed significant antitumor efficacy with negligible systemic toxicity. Thus, the findings highlight PEGylated gold nanostars as promising candidates for combined imaging and photothermal cancer therapy [73].

Jie An et al. worked with the aim of overcoming the limitations of radionuclide therapy by developing an imaging-guided multimodal strategy combining radionuclide, sonodynamic, and photothermal therapies. The innovation lies in a polydopamine-modified gold nanostar platform loaded with protoporphyrin IX and radiolabeled with ^99m^Tc/^131^I. Preclinical imaging and therapeutic studies demonstrated enhanced tumor retention and synergistic antitumor efficacy in pancreatic cancer, highlighting strong potential for future translational and clinical oncology applications [74].

Also, Hsin-Hua Hsieh et al., conducted research to overcome immunotherapy resistance in colorectal cancer by combining photothermal therapy with dendritic cell-based immunotherapy and PD-L1 blockade. In terms of radiolabeling, stability, in vitro cellular uptake, and in vivo imaging of ^111^In-atezolizumab performed using CT26 tumor-bearing mice, the triple combination showed superior antitumor efficacy, achieving 50% complete remission, prolonged survival, enhanced T-cell recruitment, and elimination of lung metastases. These results demonstrate a promising synergistic strategy for sensitizing non-responsive tumors [75].

In another study, a passive pretargeting strategy was developed by Jeroen A.C.M. Goos et al., using multivalent gold nanostars that accumulate in tumors via the EPR effect and undergo bioorthogonal click chemistry for PET imaging using an ^18^F-labeled tetrazine radiotracer. They synthesized nanostars modified with trans-cyclooctene (TCO) and evaluated in vivo click reaction with an ^18^F-labeled tetrazine radiotracer using PET imaging and biodistribution studies in tumor-bearing mice. Nanostars with directly attached TCO groups showed the highest tumor uptake and tumor-to-background ratios, while additional PEG linkers reduced targeting efficiency. Thus, the results showed the potential of nanostars as pretargeting platforms for selective delivery of radionuclides such as ^18^F for PET imaging and, in the future, therapeutic radionuclides for cancer theranostic applications [76]. Table 3 summarizes and classifies these studies.

## 5. Discussion

Gold nanostars have gained significant attention as versatile nanoplatforms for cancer theranostics, due to their unique physicochemical properties, including localized surface plasmon resonance, efficient light-to-heat conversion, strong light scattering, and tunable optical behavior within the biological window. These features make AuNSs attractive for applications in cancer diagnostics, imaging, and therapy. However, their intrinsic limitations such as insufficient tumor selectivity and surface-related hydrophobicity necessitate appropriate surface functionalization to enable their effective integration into multifunctional diagnostic and therapeutic systems [77].

To overcome these limitations, a wide range of engineered GNS-based nanocomposites has been developed, including polymer-coated, silica-supported, dendrimer-functionalized, and metal-hybrid systems. These composite structures particularly improve colloidal stability, biocompatibility, and targeting efficiency, while also supporting multifunctionality in imaging and therapeutic applications [78]. A representative example was reported by Vo-Dinh et al., who developed a GNS-based theranostic probe capable of multimodal imaging via SERS, two-photon luminescence, MRI, and CT, thereby allowing both preoperative visualization and intraoperative tumor guidance [79].

The experimental evidence summarized in this review further demonstrates the broad applicability of AuNSs across imaging modalities. Several works have established their effectiveness of AuNSs as contrast agents and multimodal imaging probes. For instance, Liu et al. showed size-dependent tumor accumulation and penetration, emphasizing the importance of controlling nanoparticle dimensions to optimize in vivo biodistribution [16]. Likewise, Hu et al. and Zhang et al. developed hybrid nanoplatforms integrating AuNSs with magnetic components, providing combined MRI, CT, and photoacoustic imaging [31,32]. These findings indicate that rational nanostructure design plays a key role in achieving efficient tumor targeting and imaging performance.

Additionally, AuNSs exhibit exceptional potential in optical imaging techniques, like SERS and fluorescence-based approaches. Minati et al. and Leyong Zeng et al. demonstrated highly sensitive intracellular imaging and real-time drug tracking provided by enhanced Raman signals at the nanostar tips [24,27]. The “lightning rod” effect associated with the sharp tips of AuNSs amplifies significantly the local electromagnetic fields, allowing ultrasensitive detection at the cellular level. Also, Wang et al. developed a smart theranostic system capable of real-time apoptosis monitoring within fluorescence signal recovery, highlighting the potential of AuNSs in self-reporting therapeutic systems [25].

Beyond their diagnosis applications, AuNSs have shown remarkable performance in therapeutic applications, especially in PTT and combinational treatments. For example, Yuan et al. demonstrated rapid and effective tumor ablation under near-infrared irradiation [7], while Liu et al. reported long-term antitumor immunity using GNS-mediated photoimmunotherapy [44]. On the other hand, multifunctional platforms integrating PTT with chemotherapy, PDT, or immunotherapy developed by Li et al. and Xia et al. saw significant improvement in therapeutic efficacy through synergistic mechanisms [48,58].

Radiotheranostic applications of gold nanostars (AuNSs) constitute an emerging field with potential for integrated imaging and therapy. Studies by Yang Liu et al. and Lin et al. have shown that ^177^Lu- and 124I-functionalized gold nanostars exhibit prolonged tumor retention and enhanced therapeutic efficacy compared to free radionuclides [70,72]. Additionally, multimodal platforms integrating PET imaging and immunotherapy, such as those developed by Cutshaw et al., enable real-time monitoring of immune responses within the tumor microenvironment [71]. These findings underscore the potential of AuNSs in advancing precision oncology through integrated diagnostic and therapeutic strategies. Despite these promising advancements, several critical challenges remain. One of the major limitations is the complexity of the biological environment, which can affect nanoparticle stability, targeting efficiency, and long-term functionality. Protein corona formation, immune clearance, and enzymatic degradation may significantly alter the physicochemical properties of AuNSs, thereby compromising their performance in vivo. As highlighted by D’Hollander et al. strategies such as surface ligand blocking can minimize these adverse effects and preserve targeting specificity [28].

Another critical challenge is the scalability and reproducibility of GNS synthesis. While several studies report controlled and relatively simple synthetic approaches, large-scale production of monodisperse and high-quality nanostars suitable for clinical use remains difficult. Additionally, although many reports indicate favorable biocompatibility, comprehensive long-term toxicity and clearance studies are still lacking, highlighting the need for more systematic evaluation.

Although gold nanostars have demonstrated considerable preclinical promise in cancer photothermal therapy and photoimmunotherapy but their translation into clinical practice is still limited. In vivo studies suggest that PEGylated or aptamer-functionalized gold nanostars can accumulate within tumor tissues; however, they also show notable retention in organs such as the liver and spleen. This indicates that their elimination is more likely mediated by the mononuclear phagocyte system rather than by rapid renal clearance [73,80,81]. In addition, once exposed to biological fluids, serum proteins can adsorb onto the nanostar surface and form a protein corona, which may influence their biodistribution, uptake by macrophages, immune recognition, and overall therapeutic performance [82,83]. Currently, clinical evidence is more advanced for other gold-based nanostructures, including gold nanoshells and gold nanorods, whereas gold nanostars remain largely confined to preclinical investigation [84].

All studies reviewed here indicate that GNS imaging and therapeutic performance results from the combined influence of their structural and surface properties. Particle size and core dimensions can affect cellular interaction and in vivo distribution, with smaller AuNSs showing better intratumoral penetration and distribution in some studies [14]. while differences in size and surface coating were also associated with changes in cellular uptake and toxicity [67]. Branch morphology also plays a crucial role. For example, AuNSs with similar core sizes but longer branches showed a red shift in plasmon resonance from 640 to 770 nm, together with improved photothermal conversion and therapeutic activity [31]. Moreover, longer and sharper branches were reported to reduce protein-corona interference and preserve HER2-specific interactions under serum-rich conditions [53]. Therefore, parameters such as particle size, core dimensions, branch length and sharpness, overall geometry, and surface chemistry collectively influence LSPR behavior, photothermal performance, cellular uptake, biodistribution, and biological response. Surface modification is another important factor in determining GNS behavior. PEGylation and other coatings have been used to improve colloidal stability, targeting, and interactions with cells [16,23,56,68]. The broader importance of PEGylation is also supported by non-gold nanostar systems; for example, Sun et al. used PEGylated Bi_2_S_3_ nanostars as mesoporous carriers for co-loading therapeutic agents and supporting controlled drug release [85], while Goos et al. investigated star-shaped polymeric nanostars functionalized with Gd^3+^, ^89^Zr, and ^177^Lu, whose tumor accumulation was mainly related to the enhanced permeability and retention (EPR) effect [86]. Although these systems are not gold nanostars, they illustrate how surface engineering and passive tumor accumulation can influence the pharmacokinetic and theranostic behavior of nanostar-shaped platforms. Thus, optimization of AuNSs for theranostic applications should consider structural geometry and surface chemistry together, since their combined effects determine optical properties, tumor accumulation, immune clearance, toxicity, and overall imaging and therapeutic performance.

Future investigations and research should address the challenges through systematic and multidisciplinary approaches. As emphasized in recent perspectives, improving nanoparticle stability, monodispersity, and optical tunability within the biological window will be crucial for advancing in clinical translation. Furthermore, to increase diagnostic accuracy and therapeutic precision, the use of combined advanced imaging techniques and computational tools is necessary [87]. Innovative methods like bioorthogonal chemistry-based tracking systems [88] offer new opportunities for real-time monitoring of nanoparticle behavior. In parallel, the incorporation of artificial intelligence into treatment planning and dosimetry is expected to play a transformative role in nanomedicine field. AI-based models can integrate imaging data, biodistribution profiles, and patient-specific parameters to optimize therapeutic strategies, improving efficacy while reducing adverse effects [89].

## 6. Conclusions

In conclusion, gold nanostars constitute a powerful and promising platform for cancer theranostics, with demonstrated capabilities in multimodal imaging, targeted treatment, and emerging radiotheranostic applications. While significant progress has been achieved in their structural design and preclinical assessment, addressing challenges related to scalability, long-term biosafety and clearance, and in vivo behavior remains essential for successful preclinical translation to reach anticipated clinical application in future. Continued interdisciplinary efforts across nanotechnology, molecular imaging, and computational medicine will be crucial to fully realize the capabilities of GNS-based systems in precision oncology.

## Figures and Tables

**Table 1:** Summary of gold nanostar-based imaging studies.

Author	Imaging Application	Scalability	Biocompatibility	In Vivo Distribution/Tumor Accumulation	Irradiation/Radiation Conditions	Principal Mechanism/Comparative Finding	Reference
Olga Bibikova et al.	OCT, Doppler OCT, confocal imaging contrast	Controlled seed-based synthesis	Living-cell visualization	Mainly cell/imaging optimization	Visible/NIR imaging wavelengths; exact conditions NR	Particle size altered plasmon peak and scattering/absorption balance, producing size-dependent contrast.	[14]
Zhang et al.	Photoacoustic imaging/PTT with green GNS	Simple surfactant-free synthesis	Good biocompatibility reported	Living cancer-cell evaluation	NIR irradiation; wavelength/power NR	Branched morphology produced NIR absorption and photothermal conversion, supporting PAI-guided PTT.	[15]
Yang Liu et al.	SERS, CT, two-photon luminescence	Size-controlled GNS probes	In vivo evaluation	Size-dependent tumor uptake studied	Clinically relevant laser conditions; exact wavelength/power NR	Smaller AuNSs showed higher intratumoral uptake/penetration; optical properties supported multimodal imaging and PTT.	[16]
Shujing Liang et al.	Photoacoustic imaging of gastric cancer stem cells	Antibody-conjugated GNS	In vitro/in vivo evaluation	Orthotopic/subcutaneous tumor accumulation	NIR laser; low power stated, exact value NR	Active CD44v6 targeting enhanced tumor localization and enabled selective PAI-guided photothermal ablation.	[17]
Vijay Raghavan et al.	Photoacoustic imaging/PTT	Chemical reduction synthesis	In vivo animal study	Tumor-bearing mouse evaluation	1064 nm laser	NIR-II excitation improved tissue penetration; GNS absorption generated PA signal and heat for tumor ablation.	[18]
Yingna Chen et al.	Photoacoustic imaging-guided PTT	Silica coating improves stability	Materials/biocompatibility focus	Imaging performance assessed	Photoacoustic/laser irradiation; wavelength NR	Silica prevented laser-induced reshaping and produced ~3-fold greater PA signal stability than uncoated AuNSs.	[19]
Yuan Zheng Wang et al.	Photoacoustic imaging-guided PTT	GNS@polyaniline synthesis	Good biocompatibility reported	Tumor targeting via HA	Photothermal irradiation; wavelength/power NR	Polyaniline enhanced photothermal conversion; HA enabled active targeting and tumor suppression at low dose.	[20]
Ping Hu et al.	CT imaging + therapy	FA-GNS preparation	Preclinical/materials evaluation	Targeting expected via folic acid	PTT + ionizing radiation; doses/wavelength NR	Gold provided CT attenuation, photothermal conversion, and radiosensitization; combined PTT/RT outperformed either treatment alone.	[21]
Lei Wang et al.	X-ray/CT contrast + PTT/PDT	Multifunctional platform	In vitro/in vivo therapy	Tumor efficacy evaluated	NIR irradiation; wavelength/power NR	AuNSs generated heat and ROS, causing DNA damage/apoptosis; high-Z gold also provided X-ray contrast.	[22]
Ana Espinosa et al.	Plasmonic thermal fingerprint imaging/therapy	Size/wavelength-dependent design	In vitro/in vivo evaluation	PC3 tumor model evaluation	Various wavelengths; exact values NR	Heating efficiency depended on GNS size and excitation wavelength, linking nanostar design to in-cell/in-tumor photothermal response.	[23]
Luca Minati et al.	SERS/confocal imaging and DOX tracking	PEGylated functionalization	Low cytotoxicity and stability reported	Mainly intracellular/cell study	Prolonged laser irradiation; wavelength/power NR	SERS tracked intracellular GNS/drug localization; laser heating triggered doxorubicin release.	[24]
Juang Wang et al.	Fluorescence apoptosis monitoring	Responsive nanocomposite	Cell-based/in vivo therapy monitoring	Tumor-targeting by folic acid	Photothermal irradiation; exact conditions NR	PTT induced apoptosis; caspase-3 activation restored fluorescence, providing treatment-response feedback.	[25]
Nergiz et al.	Raman imaging of GO-GNS nanopatches	Hybrid nano patch synthesis	Minimal cytotoxicity reported	Mainly cellular uptake	Low laser power; wavelength/power NR	Hybrid plasmonic coupling increased photothermal efficiency while Raman scattering enabled cellular imaging.	[26]
Leyong Zeng et al.	SERS imaging + PTT	Ag@Au-DTTC nanostar platform	Minimal toxicity reported	In vitro/in vivo SERS imaging	NIR laser; exact wavelength/power NR	DTTC/Au-shell design improved Raman intensity/stability and supported photothermal tumor eradication.	[27]
Antoine D’Hollander et al.	HER2-targeted darkfield/photoacoustic imaging	Surface blocking strategy	In vitro/in vivo targeting evaluated	HER2-positive tumor targeting evaluated	PA imaging; therapeutic irradiation not central/NR	Reducing protein-corona thickness preserved receptor targeting and improved tumor recognition in biological media.	[28]
Antoine D’Hollander et al.	Biocompatible tumor contrast agent for in vivo SERS	Functionalized GNS platform	Biocompatibility focus	In vivo animal tumor imaging	SERS excitation; exact wavelength NR	Systemic GNS accumulation enabled in vivo/ex vivo tumor-associated SERS signals and intracellular localization.	[29]
Joanna Depciuch et al.	Arm-size/cytotoxicity and photothermal effect	Morphology control addressed	Cytotoxicity evaluated	In vitro only	Laser irradiation; wavelength NR; LSPR shifted 640 → 770 nm	Longer branches red-shifted LSPR and increased heat generation; longest-branched AuNSs reduced viability by ~75%.	[30]
Luyun Zhang et al.	MRI-guided PTT	MOF-encapsulated single GNS	Preclinical evaluation	Targeting peptide improves localization	808 nm, low-power laser	MOF enabled MRI; ZD2 improved TNBC targeting; GNS photothermal conversion (~40.5%) enabled guided PTT.	[31]
Yong Hu et al.	MRI/CT/photoacoustic imaging	Fe_3_O_4_@Au core–shell nanostars	Biocompatibility evaluated	Tumor-targeted multimodal imaging	NIR irradiation; exact conditions NR	Magnetic core and high-Z/plasmonic Au shell enabled multimodal imaging; FA supported receptor targeting and PTT.	[32]
Peng Ning et al.	Multimodal tracking of photosensitive stem cells	Functionalized platform	Cell-based evaluation	Tracking-oriented, mainly preclinical	PTT irradiation; wavelength/power NR	MSC carriage enhanced tumor delivery; 3D PAI identified peak accumulation at 24 h to guide treatment timing.	[33]
Hyojin Lee et al.	Lysosome-targeting intracellular evaluation	Nanoconstruct design	In vitro cancer-cell study	No in vivo distribution focus	No external irradiation required in described mechanism	HER2-mediated endocytosis and lysosomal trafficking promoted HER2 degradation, apoptosis, and cell-cycle arrest.	[34]
Asrin Pakravan et al.	Chemo-photothermal imaging-related nanostars	Polymer-coated nanostar/nanocage system	In vitro breast cancer cells	No in vivo distribution	NIR irradiation; exact wavelength NR; ΔT ~23–27 °C	Hollow AuNSs outperformed gold nanocages photothermally; combined DOX + PTT produced up to 90% mortality and 97% apoptosis.	[35]
Tan et al.	NIR fluorescence/photoacoustic imaging	Multifunctional CD133-targeted probe	In vitro/in vivo evaluation	Tumor targeting confirmed	NIR irradiation; exact wavelength/power NR	One NIR trigger combined heat, singlet oxygen, and drug release; CD133 targeting improved tumor localization.	[36]
Kazuki Sato et al.	EV-GNS hybrid tracking/therapy relevance	EV-GNS assembly	In vitro evaluation	No strong in vivo distribution focus	Laser-responsive PTT; exact conditions NR	EV-GNS assembly preserved EV biology while improving photothermal performance and laser-triggered cytotoxicity.	[37]

**Table 2:** Summary of gold nanostar-based therapy studies.

Author	Therapy Application	Scalability	Biocompatibility	In Vivo Distribution/Tumor Accumulation	Irradiation/Radiation Conditions	Principal Mechanism/Comparative Finding	Reference
Hamed Aram	Wireless NIR photothermal therapy for brain tumors	Advanced engineered system	In vivo mouse study	Tumor-specific diffusion/heating	Wireless control of wavelength, power, and photothermal intensity; exact values NR	Localized GNS heating enabled on-demand minimally invasive tumor ablation with adjustable optical parameters.	[43]
Yang Liu	SYMPHONY photoimmunotherapy for GBM	Preclinical GNS platform	In vivo therapeutic response	Tumor model response/rechallenge	PTT irradiation; exact conditions NR	PTT + checkpoint blockade generated durable responses, survival benefit, and resistance to tumor rechallenge.	[44]
Yuting Zhu	GNS/MWCNT photothermal therapy	Controlled two-step synthesis	High biocompatibility reported	Mainly in vitro	808 nm irradiation	Plasmonic/coupling effects increased photothermal conversion and cancer-cell ablation at low concentrations.	[45]
Meijun Zhao	Nanozyme-enhanced PTT	One-step green synthesis	In vivo evaluation	Photoacoustic-guided tumor ablation	Photothermal irradiation; exact wavelength/power NR	Glucose oxidation/H_2_O_2_ decomposition generated hydroxyl radicals; heating amplified catalytic activity for synergistic tumor ablation.	[46]
Juan F. Ramirez Henao	Chemo-photothermal AuNS@POM	Hybrid nanocomposite synthesis	Cancer-cell evaluation	Mainly in vitro	NIR irradiation; exact conditions NR	POM supplied intrinsic anticancer/redox activity while AuNSs supplied photothermal heating.	[47]
Rong-Tian Li	HA-targeted PDT/PTT	Multifunctional nanosystem	In vivo tumor suppression	CD44-targeted delivery	Light-triggered PDT/PTT; exact wavelength NR	BD3 generated singlet oxygen and AuNSs generated heat; HA targeting enhanced delivery and combined apoptotic/necrotic killing.	[48]
Bridget M. Crawford	PTT of inflammatory breast cancer emboli	Plasmonic GNS platform	In vivo animal study	Tumor emboli targeting/effect	808 nm continuous-wave laser; different power densities	GNS penetration into embolic cores enabled photothermal ablation independent of receptor status/drug resistance.	[49]
Ziwei Li	Ce6/PDA-GNS PDT/PTT	Coated/conjugated GNS	Method/materials evaluation	Lung metastasis inhibition focus	NIR laser; exact conditions NR	PDA enhanced photothermal/PA performance; Ce6 produced singlet oxygen; combination inhibited primary tumor and lung metastasis.	[50]
Chunhui Wu	Graphene/GNS PDT/PTT	Graphene-GNS nanoplatform	Good biocompatibility reported	In vivo tumor eradication	Single laser wavelength; exact value NR	Spectral matching co-activated heat and ROS; lysosomal/mitochondrial disruption contributed to synergistic tumor eradication.	[51]
Lin Zhang	CT imaging + catalase-enhanced PDT/PTT	Intelligent GNS platform	In vivo animal study	Tumor imaging/therapy	Photo-controlled PDT/PTT; exact wavelength NR	Catalase relieved hypoxia, Ce6 supplied PDT, AuNSs supplied PTT/CT contrast, and cRGDyK improved targeting.	[52]
Irfan Shafi Malik	Aptamer-targeted therapy	Branch morphology control	Serum/protein corona relevance	Mainly cell/targeting study	No irradiation condition central to reported comparison	Longer/sharper branches reduced corona interference and preserved receptor interactions, improving targeted cytotoxicity.	[53]
Mohanna Etemad	Computational PTT optimization	Modeling study	No biological toxicity data	No distribution data	NIR laser; treatment time, intensity, radius, temperature modeled	Defined parameter relationships needed to destroy tumor while avoiding surface overheating; no biological toxicity data.	[54]
Amir Soleimany	Two-photon PDT/PTT	GNS/GQD nanohybrid	Method/materials study	Not tumor distribution-focused	Low-power pulsed single-laser irradiation; exact wavelength NR	GNS heating plus plasmon-enhanced ROS from GQDs produced synergistic PDT/PTT.	[55]
Shouju Wang	pH-controlled uptake + PTT	Mixed-charge PEGylated GNS	In vitro cell study	In vitro and in vivo tumors	PTT irradiation; exact conditions NR	Acidic pH switched cellular affinity/uptake on, increasing tumor accumulation and PTT while limiting interaction at pH 7.4.	[56]
Min Woo Kim	Platelet-like GNS cancer therapy	Biomimetic GNS platform	Immune evasion evaluated	Tumor therapy relevance	NIR irradiation; exact conditions NR	Platelet membrane enhanced tumor targeting; RBC membrane reduced macrophage clearance; NIR triggered drug release and PTT.	[57]
Fangfang Xia	PA/NIR imaging-guided PDT/PTT	BSA-GNS multifunctional probe	Favorable biocompatibility	MMP2-targeted tumor imaging	Single light source; exact wavelength NR	MMP2 targeting improved tumor localization; IR-780/GNS combination generated ROS and heat for dual therapy.	[58]
Yang Li	Integrin-targeted PTT for HCC	PDA-coated targeted GNS	Cell-based evaluation	HepG2 HCC cells and mouse tumors	NIR-based PTT; absorption peak ~822 nm; irradiation setting NR	alpha-v-beta-3 targeting increased uptake; photothermal stress induced mitochondrial/lysosomal/autophagy-related death and tumor suppression.	[59]
Ericka Chorniak	Photoimmunotherapy	Preclinical platform	In vivo immune response monitored	Distant tumor immune infiltration studied	PTT irradiation; exact conditions NR	Local PTT combined with checkpoint inhibition increased systemic immune-cell infiltration and antitumor response.	[60]
Shujing Liang	Immune cell-mediated PTT/PDT delivery	Complex cell-drug platform	In vivo multimodal evaluation	Enhanced intratumoral targeting	NIR irradiation; exact conditions NR	CIK cells improved intratumoral delivery; trastuzumab targeted HER2; GNS/ICG provided combined PTT/PDT and immune activation.	[61]
Peng Xu	Light-triggered CPT delivery + PTT	Cucurbituril-stabilized platform	Therapy-focused	Targeted/light-triggered release	NIR irradiation; exact conditions NR	NIR simultaneously triggered CPT release and GNS heating, creating chemo-photothermal synergy.	[62]
Yingke Hou	CPT prodrug + radiosensitization + PTT	Multifunctional hierarchical platform	Therapy study	CD44-targeting and intracellular tracking	Ionizing radiation + mild PTT; dose/wavelength NR	High-Z gold increased radiation energy deposition; mild PTT reduced hypoxia; prodrug/targeting added chemotherapy and immunogenic cell death.	[63]
Yanlei Liu et al.	CaCO_3_-triggered drug release + PDT/PTT	Encapsulated GNS system	In vivo animal study	Tumor-triggered release/effect	NIR irradiation; exact conditions NR	CaCO_3_ acted as pH-responsive reservoir/protection; ICG supplied PDT/imaging and AuNSs supplied PTT.	[64]
Ping Wei et al.	CT/PTT/gene silencing	Dendrimer-stabilized GNS	In vivo animal study	Tumor imaging/therapy	NIR irradiation; exact conditions NR	RGD improved targeting; combined GNS photothermal ablation with VEGF gene silencing, while Au supported CT/thermal imaging.	[65]

**Table 3:** Summary of studies using gold nanostar in radiotheranostic application.

Study/Classification	Radionuclide/Radiation Type	Labeling Chemistry	GNS Architecture	Tumor Model	Imaging Modality	Therapeutic Modality	Tumor Retention/Biodistribution	Therapeutic/Diagnostic Outcome
An et al. [74] Combined imaging-guided radionuclide/SDT/PTT	^99m^Tc; γ */^131^I; β^−^/γ *	Polydopamine-modified GNS platform loaded with protoporphyrin IX and radiolabeled with ^99m^Tc/^131^I; exact labeling chemistry NR	PDA-modified GNS + protoporphyrin IX	Preclinical pancreatic tumor model; exact model details NR	Imaging-guided treatment; specific imaging modality NR in the manuscript	Combined radionuclide therapy + sonodynamic therapy + photothermal therapy	Enhanced tumor retention, quantitative values/duration NR	Synergistic antitumor efficacy reported
Chen et al. [73] Radionuclide tracking + GNS photothermal therapy	^111^In; γ (electron-capture) *	^111^In-DTPA-labeled PEGylated AuNSs used as a radioactive surrogate	HEPES-synthesized PEGylated AuNSs	SKOV-3 xenograft tumor model	microSPECT/CT for noninvasive biodistribution tracking	Photothermal therapy; ^111^In used for tracking rather than radionuclide therapy	Radioactive signal correlated strongly with gold content; quantitative tumor-retention duration NR	Significant antitumor efficacy after PTT
Cutshaw et al. [71] Radionuclide imaging/treatment-response monitoring	^64^Cu; β^+^ (PET) *	NOTA-chelated ^64^Cu; AuNSs additionally functionalized with Raman reporters and immune-targeting antibodies	Multimodal gold nanostars (MGNs)	CT26 immunologically “hot” and 4T1 immunologically “cold” murine tumors	ImmunoPET + SERS; findings validated by immunofluorescence, ELISA, and flow cytometry	No radionuclide therapy: imaging used to monitor response to anti-PD-L1 + anti-CD47 immunotherapy	Immune-cell recruitment within tumors was tracked; quantitative GNS tumor-retention data NR	Enabled in vivo tracking of CD8^+^ T cells and NK cells and prediction of treatment response
Goos et al. [76] Pretargeted radionuclide imaging	^18^F; β^+^ (PET) *	Bioorthogonal pretargeting: TCO-functionalized AuNSs reacted in vivo with an ^18^F-labeled tetrazine radiotracer; direct TCO attachment and PEG-linker designs compared	Multivalent TCO-functionalized gold nanostars	Tumor-bearing mice; exact tumor model NR in the manuscript	PET imaging + biodistribution studies	future therapeutic radionuclide delivery proposed	Directly attached TCO groups gave the highest tumor uptake and tumor-to-background ratios; additional PEG linkers reduced targeting efficiency	Demonstrated feasibility of passive pretargeting and selective ^18^F delivery for PET
Hsieh et al. [75] Indirect/adjunct nuclear imaging with GNS-based therapy	^111^In; γ (electron-capture) *	^111^In-labeled atezolizumab; radionuclide is attached to the antibody, not directly to the GNS	Gold nanostars used for PTT in a combined immunotherapy strategy	CT26 tumor-bearing mouse model (colon carcinoma)	In vivo imaging of ^111^In-atezolizumab; specific nuclear imaging modality NR	GNS-mediated PTT + dendritic-cell immunotherapy + PD-L1 blockade	NR	50% complete remission, prolonged survival, enhanced T-cell recruitment, and elimination of lung metastases reported
Lin et al. [72] Direct therapeutic radiotheranostic/combined therapy	^177^Lu; β^−^/γ *	DTPA-mediated ^177^Lu labeling of PEGylated AuNSs (^177^Lu-DTPA-pAuNS); RadioTLC and EDTA stability testing described	PEGylated gold nanostars conjugated with DTPA	Orthotopic head and neck squamous cell carcinoma (HNSCC) model	Biodistribution and dosimetry evaluation; specific imaging modality	^177^Lu-based brachytherapy; combination with photothermal therapy	Prolonged tumor retention up to 15 days; free ^177^Lu-DTPA was undetectable after 24 h	Significant tumor-growth suppression; therapeutic effect further enhanced by PTT
Liu et al. [69] Direct radionuclide therapy	^211^At; α-particle therapy (explicitly stated)	Rapid ^211^At labeling of AuNSs (~100% within 1 min); >99% activity retained after 24 h in serum. Exact chemical linkage: NR.	^211^At-labeled gold nanostars	U87MG human glioma xenograft mouse model	Biodistribution assessment; dedicated imaging modality NR	Targeted α-particle therapy; intratumoral administration	Tumor-retention duration NR. Minimal deastatination; low thyroid (0.44–0.64% ID) and stomach (0.21–0.49% ID) uptake.	Significant suppression of tumor growth
Liu et al. [70] Radionuclide-associated imaging	^124^I; β^+^/γ *	^124^I-GNS nanoprobe; exact radioiodination chemistry NR	Systemically administered ^124^I-labeled GNS	Murine orthotopic glioma model	Optical imaging and TEM; radionuclide imaging modality	None; imaging-focused study	High tumor-to-normal uptake ratio; extravasation from tumor vasculature, interstitial penetration, and cellular internalization.	Sensitive tumor detection and tracking; lesions as small as ~0.5 mm reported

Note: * Radiation types, NR: not reported.

## Data Availability

No new data were created or analyzed in this study. Data sharing is not applicable to this article.

## Abbreviations

The following abbreviations are used in this manuscript:

AuNPsp	gold nanoparticle spherical
AuNPst	gold nanoparticle star
AuNPr	gold nanoparticle rod
Au@PDA-RGD NPs	RGD peptide-conjugated polydopamine-coated gold nanostars
AuNS-TCS	thiolated chitosan-coated gold nanostars
CT	computed tomography
Ce6	chlorin e6
CB [7]	cucurbit [7] uril
CPT	camptothecin
DTTC	3,3′-diethylthiatricarbocyanine
EM	electromagnetic
ECL	enhanced electrochemiluminescence
EV-GNS	extracellular vesicle–gold nanostar
EPR	enhanced permeability and retention
AuNSs	gold nanostars
GNS-FA	folic acid-functionalized gold nanostars
g-CN	graphitic carbon nitride
HApt-AuNS	anti-HER2 aptamer-functionalized gold nanostars
HAuNSs@Dox-Pol	doxorubicin-loaded hollow gold nanostars
HCC	hepatocellular carcinoma
HEPES	2-[4-(2-hydroxyethyl)-1-piperazinyl] ethanesulfonic acid
HNSCC	head and neck squamous cell carcinoma
IBC	inflammatory breast cancer
LSPR	localized surface plasmon resonance
Micro STEM	micro scanning transmission electron microscopy
MRI	magnetic resonance imaging
MR	magnetic resonance
MGNs	multimodal gold nanostars
MSCs	mesenchymal stem cells
MAuNSs	mesoporous silica-coated gold nanostars
NIR	near infrared
NK	natural killer
PDT	photodynamic therapy
PTT	photothermal therapy
PAI	photoacoustic imaging
PET	positron emission tomography
RECIST	Response Evaluation Criteria in Solid Tumors
ROS	reactive oxygen species
R/P-cGNS	red blood cell and platelet membrane-coated gold nanostars loaded with curcumin
RGD-Au DSNSs	RGD-modified dendrimer-stabilized gold nanostars
SERS	surface-enhanced Raman scattering
SYMPHONY	synergistic immuno-photothermal nanotherapy
SEAD	selected area diffraction
TCO	*trans*-cyclooctene
TAT	targeted alpha-particle therapy
TNBC	triple-negative breast cancer
X-Ray	X-ray diffraction
ZIF-8	zeolitic imidazole framework-8
